# Seizure Susceptibility and Sleep Disturbance as Biomarkers of Epileptogenesis after Experimental TBI

**DOI:** 10.3390/biomedicines10051138

**Published:** 2022-05-14

**Authors:** Pedro Andrade, Leonardo Lara-Valderrábano, Eppu Manninen, Robert Ciszek, Jesse Tapiala, Xavier Ekolle Ndode-Ekane, Asla Pitkänen

**Affiliations:** A. I. Virtanen Institute for Molecular Sciences, University of Eastern Finland, P.O. Box 1627, FI-70211 Kuopio, Finland; pedro.andrade@uef.fi (P.A.); leonardo.laravalderrabano@uef.fi (L.L.-V.); eppu.manninen@uef.fi (E.M.); robert.ciszek@uef.fi (R.C.); jesseta@student.uef.fi (J.T.); xavier.ekollendode-ekane@uef.fi (X.E.N.-E.)

**Keywords:** biomarker, hypnogram, lateral fluid percussion, pentylenetetrazol, post-traumatic epilepsy

## Abstract

Objectives: We investigated whether seizure susceptibility increases over weeks–months after experimental traumatic brain injury (TBI), and whether seizure susceptibility in rats predicts the development of post-traumatic epilepsy (PTE) or epileptiform activity. We further investigated whether rats develop chronic sleep disturbance after TBI, and whether sleep disturbance parameters—alone or in combination with pentylenetetrazol (PTZ) test parameters—could serve as novel biomarkers for the development of post-traumatic epileptogenesis. Methods: TBI was induced in adult male Sprague-Dawley rats with lateral fluid-percussion injury. Sham-operated experimental controls underwent craniectomy without exposure to an impact force. Seizure susceptibility was tested with a PTZ test (30 mg/kg, intraperitoneally) on day (D) 30, D60, D90, and D180 after TBI (*n* = 28) or sham operation (*n* = 16) under video electroencephalogram (vEEG). In the 7th post-injury month, rats underwent continuous vEEG monitoring to detect spontaneous seizures and assess sleep disturbances. At the end of the experiments, rats were perfused for brain histology. Results: In the TBI group, the percentage of rats with PTZ-induced seizures increased over time (adjusted *p* < 0.05 compared with D30). Combinations of three PTZ test parameters (latency to the first epileptiform discharge (ED), number of EDs, and number of PTZ-induced seizures) survived the leave-one-out validation for differentiating rats with or without epileptiform activity, indicating an area under the receiver operating curve (AUC) of 0.743 (95% CI 0.472–0.992, *p* = 0.05) with a misclassification rate of 36% on D90, and an AUC of 0.752 (95% CI 0.483–0.929, *p* < 0.05) with a misclassification rate of 32% on D180. Sleep analysis revealed that the number of transitions to N3 or rapid eye movement (REM) sleep, along with the total number of transitions, was increased in the TBI group during the lights-on period (all *p* < 0.05). The sleep fragmentation index during the lights-on period was greater in the TBI rats than in sham-operated rats (*p* < 0.05). A combination of sleep parameters showed promise as diagnostic biomarkers of prior TBI, with an AUC of 0.792 (95% CI 0.549–0.934, *p* < 0.01) and a misclassification rate of 28%. Rats with epilepsy or any epileptiform activity had more transitions from N3 to the awake stage (*p* < 0.05), and the number of N3–awake transitions differentiated rats with or without epileptiform activity, with an AUC of 0.857 (95% CI 0.651–1.063, *p* < 0.01). Combining sleep parameters with PTZ parameters did not improve the biomarker performance. Significance: This is the first attempt to monitor the evolution of seizure susceptibility over months in a well-described rat model of PTE. Our data suggest that assessment of seizure susceptibility and sleep disturbance can provide diagnostic biomarkers of prior TBI and prognostic biomarkers of post-traumatic epileptogenesis.

## 1. Introduction

Approximately 70 million individuals are estimated to suffer a TBI each year [1]. TBIs account for 10% to 20% of acquired structural epilepsy [2,3]. The risk of post-traumatic epilepsy (PTE) increases as the severity of the TBI increases—approximately 2–4-fold after mild, 8-fold after moderate, and 16-fold after severe TBI [4]. Despite over 20 favorable preclinical proof-of-concept trials investigating more than a dozen different interventions in animal models of PTE, there are currently no available treatments for patients at risk of epileptogenesis after TBI [5]. A major obstacle to the development of therapies is the lack of diagnostic biomarkers of epileptogenesis that could be used to both stratify subjects for preclinical and clinical anti-epileptogenesis studies, and decrease trial costs [5,6,7].

Epileptogenesis refers to the development and extension of tissue capable of generating spontaneous seizures, resulting in (a) the development of an epileptic condition and/or (b) progression of epilepsy after it is established [8]. After severe TBI in humans, approximately 60% of TBI patients who eventually develop epilepsy will receive a PTE diagnosis within 1 year (and 80% within 2 years) after the TBI [4,9]. After severe lateral-fluid-percussion-induced TBI in rats—a well-characterized model of human TBI—approximately 15% of rats develop epilepsy within 3 months, 25% within 6 months, and 50% within 12 months [5]. Consistent with this finding, preclinical studies have demonstrated that post-injury molecular and cellular alterations linked to epileptogenesis progress within hours to days to weeks to months, in spatially and temporally orchestrated waves, suggesting a gradual increase in seizure susceptibility. However, epileptogenesis varies between individuals, depending on the type, location, and severity of the precipitating injury, as well as subject-related factors, such as genetics and post-injury infections, thereby increasing the complexity of the epileptogenic process, and emphasizing the need for personalized markers of risk determination during the course of the epileptogenic process [10,11].

To date, the results of cross-sectional studies have identified promising blood, imaging, and electroencephalogram (EEG) biomarkers of epileptogenesis after TBI [5,12]. However, limited data are available on the changes that occur in proposed biomarker levels over the course of epileptogenesis [5]. In animal models, seizure-susceptibility tests using chemoconvulsants or electrical stimulation offer rather noninvasive tools that can be used repeatedly to identify test parameters that best monitor the evolution of excitability within the epileptogenic neuronal network [13]. Similarly, in humans, recent developments in transcranial magnetic stimulation have raised expectations for its application to noninvasively monitor network reorganization and progression of cortical excitability after TBI [14,15].

Administration of a subconvulsive dose of the chemoconvulsant pentylenetetrazol (PTZ) is widely used to evaluate seizure susceptibility in genetically modified mice, inbred mice and rats with spontaneous seizures, and animals exposed to various epileptogenic brain injuries, including TBIs, as well as to identify antiseizure medications [16]. The PTZ test is also used to identify epileptogenic cortical areas in drug-refractory patients with PTE as part of the epilepsy surgery evaluation [17]. PTZ rapidly penetrates the blood–brain barrier, and its elimination half-life in rats is approximately 2 to 3 h [18,19,20]. All but one study of PTZ-induced seizure susceptibility in TBI models reported to date have been cross-sectional, using a single administration of PTZ from 4 days to 12 months after TBI. A study by Saraiva et al. [21] injected PTZ at 4 d and 8 d after TBI. Thus, no long-term studies are available that monitored the evolution of seizure susceptibility in individual animals after TBI.

Here, we tested the hypothesis that PTZ-induced seizure susceptibility increases over a period of weeks to months after experimental TBI. We further evaluated whether a clear increase in seizure susceptibility observed in rats could predict the development of PTE or epileptiform activity. In addition, as late unprovoked seizures and epileptiform activity typically occur at the transition between N3 and rapid eye movement (REM) sleep [22], we investigated whether parameters indicating TBI-induced sleep disturbance—alone or in combination with PTZ test parameters—could serve as sensitive and specific prognostic biomarkers for epileptogenesis.

## 2. Methodology

**Figure 1:**
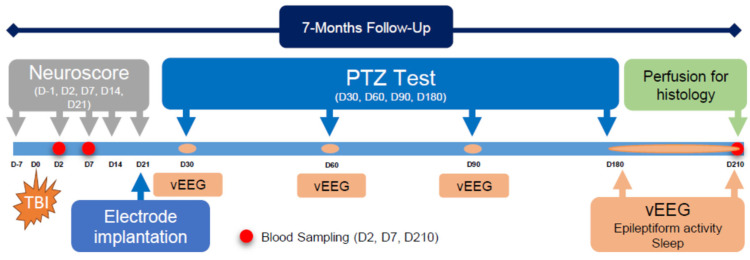
Study design: Epileptogenesis was triggered by lateral-fluid-percussion-induced traumatic brain injury (TBI, injury day (D) 0). The composite neuroscore test was performed at baseline (before TBI), and then on D2, D7, D14, and D21 post-TBI. Tail vein blood was sampled on D2 (48 h), D7, and D210 after TBI (data not shown). Epidural cortical electrodes were implanted on D21. Seizure susceptibility was assessed with the pentylenetetrazol (PTZ, 25 mg/kg, intraperitoneally) test on D30, D60, D90, and D180. Video electroencephalography (vEEG) was monitored starting 24 h before, during, and for 24 h after each PTZ test. During the 7th post-injury month (D180–D210), animals were continuously monitored via vEEG to detect spontaneous seizures and record sleep. At the end of the monitoring, animals were perfused for histological analysis.

The study design is summarized in Figure 1.

### 2.1. Animals

Adult male Sprague-Dawley rats (*n* = 50, Harlan, The Netherlands) were used (weight 321 ± 27 g on the day of surgery). Rats were housed in individual cages and maintained in a controlled environment (12 h light/12 h dark cycle, temperature 22 ± 1 °C; humidity 50–60%). Water and food were available ad libitum. Experiments were approved by the Committee for the Welfare of Laboratory Animals of the University of Eastern Finland. All of the experiments were conducted according to the European Community Council Directives 2010/63/EU.

### 2.2. Induction of Lateral Fluid-Percussion Injury

**Figure 2:**
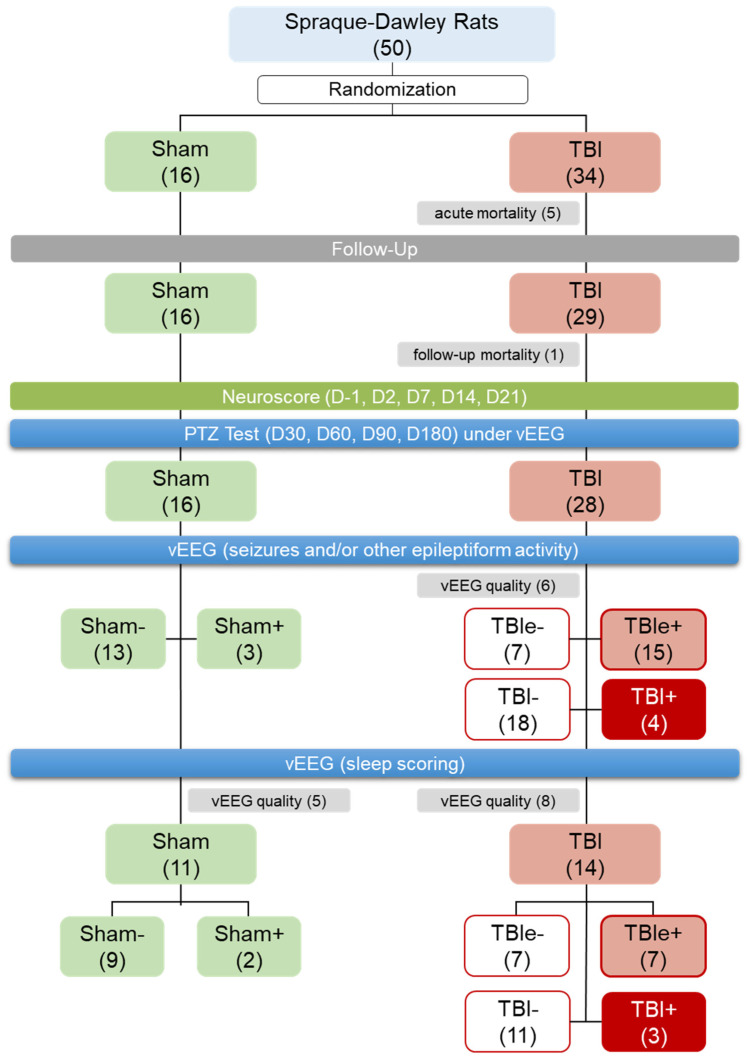
Randomization and study flow: We aimed at having at least 20 rats with traumatic brain injury (TBI) and 10 sham-operated experimental controls to complete the 7-month follow-up. In the TBI group, we expected 25% acute mortality (due to impact) and a 25% follow-up exclusion rate (mostly related to mortality in the pentylenetetrazol (PTZ) test or loss of the electrode headset). In the sham-operated group, we expected 0% acute mortality and a 15% follow-up exclusion rate (due to loss of electrode headset). Altogether, 50 rats were randomized into the sham-operation (*n* = 16) or lateral fluid-percussion injury (*n* = 34) groups. Acute mortality in the TBI group was 15% (5/34), and follow-up mortality was 3% (1/29). Consequently, 16 sham-operated rats and 28 rats with TBI completed the PTZ testing and 1-month video electroencephalogram (vEEG) monitoring. Of the 28 rats with TBI, 6 had an EEG of insufficient quality, and were excluded from the analysis of epileptiform activity. Of the remaining 22 rats with TBI, 15 showed epileptiform activity in EEG (TBIe+) and 7 did not (TBIe−). Four of the 22 TBI rats (i.e., 18%) had unprovoked seizures (TBI+, i.e., post-traumatic epilepsy) and 18 did not (TBI−). Of the 16 rats in the sham group and 28 in the TBI group, 11 (69%) and 14 (50%), respectively, had an EEG of sufficient quality for sleep scoring, and were included in the sleep analysis.

A total of 50 rats were randomized to the sham operation (*n* = 16) or TBI (*n* = 34) groups, as summarized in Figure 2. TBI was induced by lateral fluid-percussion injury (FPI), as previously described in [23]. Briefly, the animals were anesthetized with an intraperitoneal injection of a solution (6 mL/kg) containing sodium pentobarbital (58 mg/kg), magnesium sulfate (127 mg/kg), propylene glycol (43%), and absolute ethanol (11%), and placed in a Kopf stereotactic frame (David Kopf Instruments, Tujunga, CA, USA). The skull was exposed with a midline skin incision, and the periosteum was extracted. The left temporal muscle was gently detached from the lateral ridge. A circular craniectomy (Ø 0.5 mm) was performed over the left parietal cortex midway between the lambda and the bregma (center coordinates: 4.5 mm posterior and 2.5 mm lateral to the bregma), leaving the dura intact. After placing a modified Luer-lock cap over the craniectomy, its edges were sealed carefully with tissue adhesive (3M Vetbond, 3M Deutschland GmbH, Germany) and covered with dental acrylate (Selectaplus CN, Dentsply DeTrey GmbH, Dreieich, Germany), and the cap was filled with sterile 0.9% NaCl. Lateral FPI was induced 90 min after administration of the anesthetic cocktail. The rat was then connected to a fluid-percussion device (AmScien Instruments, model FP302, Richmond, VA, USA) via the Luer-lock fitting. The impact was set at 3.0 atm to produce severe TBI (expected acute mortality of ~20%). The 16 sham-operated experimental controls underwent the same procedures, excluding the trauma induction.

### 2.3. Electrode Implantation

**Figure 3:**
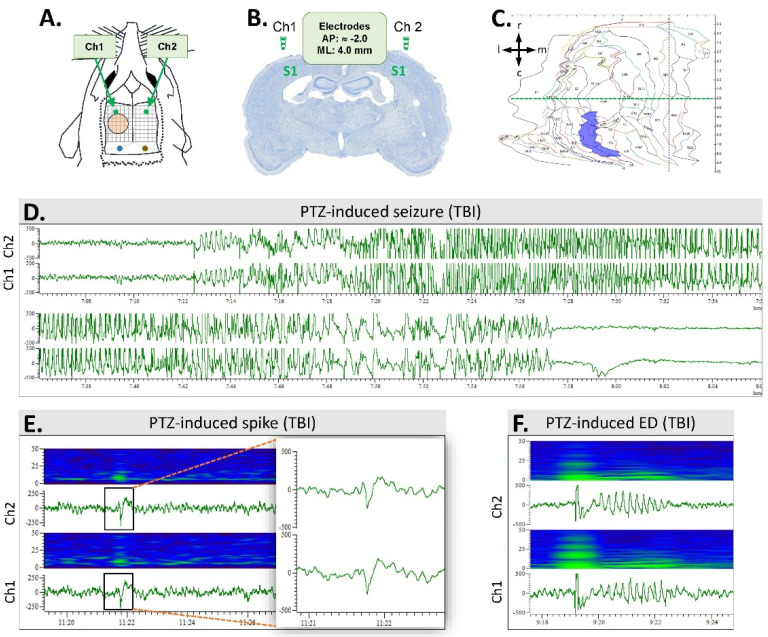
Electrode montage and pentylenetetrazol (PTZ)-induced epileptiform activities: (**A**) Electroencephalogram (EEG) was monitored using 2 epidural screw electrodes (Ch1 and Ch2) implanted bilaterally, rostral to the level of the anterior edge of the craniotomy (orange circle). Reference (blue) and ground (brown) electrodes were placed above the cerebellum. Grid 1 mm × 1 mm. (**B**) A coronal thionin-stained section from the level of epidural electrodes above the S1 cortex of a rat with TBI (case #11). The level is slightly rostral to the beginning of the lateral-fluid-percussion-induced cortical lesion. Note the enlarged lateral ventricles. (**C**) A computer-generated unfolded cortical map of case #11, demonstrating the location and area (7.72 mm^2^) of the cortical lesion (violet). The horizontal dashed green line indicates a bregma level of −2.0 (corresponding to panel B, taken approximately 1 mm rostral to the beginning of the cortical lesion). Arrows indicate the orientation of the cortical unfolding (r, rostral; c, caudal; m, medial; l, lateral). (**D**) A PTZ-induced seizure in a TBI rat (case #9). (**E**) A heatmap and an EEG trace showing a PTZ-induced spike in a TBI rat (case #9). The insert on the right shows the spike enlarged. Time scale is on the *x*-axis and voltage (300 μV) on the *y*-axis. (**F**) A heatmap and an EEG trace showing a PTZ-induced epileptiform discharge (ED; case #45).

On day (D) 21 after TBI or sham operation (D0 was marked as the day of injury), rats were anesthetized as described above and inserted into the stereotaxic apparatus. Two cortical stainless steel screw electrodes (E363/20 Plastics One Inc., Roanoke, VA, USA) were inserted over the parietal cortex, targeting the S1 area (AP −2.0, ML 4.0)—one rostral to the craniotomy, and the other at the corresponding coordinates contralaterally (Figure 3A,B). Two screw electrodes were inserted into the skull bilaterally over the cerebellum to serve as reference and ground electrodes. The electrodes were connected to a plastic pedestal (Plastics One, Inc., Roanoke, VA, USA) that was cemented onto the skull with dental acrylic.

### 2.4. Video Electroencephalogram (vEEG) Recording

Long-term vEEG monitoring was performed to (a) assess the induced epileptiform activity in the PTZ seizure-susceptibility tests on D30, D60, D90, and D180, (b) detect unprovoked seizures, i.e., diagnose PTE during the 7th post-injury month, (c) record other types of epileptiform activity during the 7th post-injury month, and (d) monitor sleep during the 7th post-injury month.

Details of the methodology were described previously in [24]. Briefly, the animal was placed into a Plexiglas cage, where it could move freely (1 rat per cage). Electrical brain activity was monitored using the Nervus EEG Recording System connected to a Nervus magnus 32/8 Amplifier (Taugagreining, Reykjavik, Iceland), sampled at a rate of 2 kHz, and filtered (high-pass filter: 1 Hz; low-pass filter: 70 Hz). The behavior of the animal was recorded using a WV-BP330/GE Video Camera (Panasonic, Kadoma, Japan) positioned above the cages and connected to an SVT-N72P Time-Lapse VCR (Sony, Tokyo, Japan) and a PVM-145E Video Monitor (Sony). A wide-angle lens was used to simultaneously videotape 10 animals. WFL-II/LED15W infrared light (Video Technical, GmbH, Nurenberg, Germany) was used at night to allow for continuous 24 h/day video monitoring.

### 2.5. Composite Neuroscore

Deficits in the somatomotor and vestibular functions were assessed by the composite neuroscore test, as previously described in [25]. Testing was performed 1 day (-1D) before TBI (baseline), and on D2, D7, D14, and D21 post-TBI (Figure 1). As outcome measures, the rats were scored from 0 (severely impaired) to 4 (normal) on an ordinal scale for each of the following 7 parameters: (1) left and right forelimb flexion during tail suspension (2 parameters), (2) left and right hindlimb flexion while the rat was gently pulled back by the tail along the table surface (2 parameters), (3) ability to resist left and right lateral pulsion (2 parameters), and (4) ability to stand on an inclined angle board. The maximum score was 28.

### 2.6. Pentylenetetrazol Seizure-Susceptibility Test

To monitor the evolution of post-TBI seizure susceptibility, rats underwent a PTZ seizure-susceptibility test on D30, D60, D90, and D180 after TBI. Rats were connected to the vEEG system 24 h before the PTZ test, and monitoring was continued for 24 h after the test, after which the rats were disconnected from the monitoring system (Figure 1). A subconvulsant dose of PTZ (1,5-pentamethylenetetrazole, 98%, Sigma-Aldrich YA-Kemia Oy, Finland, 25 mg/kg) was administered intraperitoneally in 0.9% NaCl (2 mL/kg) [23]. Immediately following the PTZ injection, the rats were placed back into their cages, and vEEG monitoring was continued. On each testing day, the following outcome measures were assessed from a 60-min EEG epoch after the saline/PTZ injection: (1) latency to the first spike, (2) latency to the first epileptiform discharge (ED), (3) latency to the 1st, 2nd, … nth seizure, (4) number of epileptiform spikes (per 60 min after the saline/PTZ injection; spikes related to seizures or EDs were excluded), (5) number of EDs, (6) number of seizures, (7) duration of each seizure, (8) percentage of rats expressing a seizure after each PTZ injection, and (9) behavioral severity of each seizure [26] (Figure 3).

A seizure was defined as a high-amplitude rhythmic discharge with frequency and amplitude modulation that clearly represented an abnormal EEG pattern (repetitive spikes, spike-and-wave discharges, poly-spike-and-wave discharges, or slow waves) and lasted at least 10 s. An electrographic ED was defined as a rhythmic high-amplitude discharge, containing a burst of slow waves, spike-and-wave, and/or poly-spike-and-wave components, and lasting < 10 s. Spikes were defined as high-amplitude electrographic elements with a duration between 20 and 70 ms.

### 2.7. Analysis of Unprovoked Seizures and Other Epileptiform Activity

**Figure 4:**
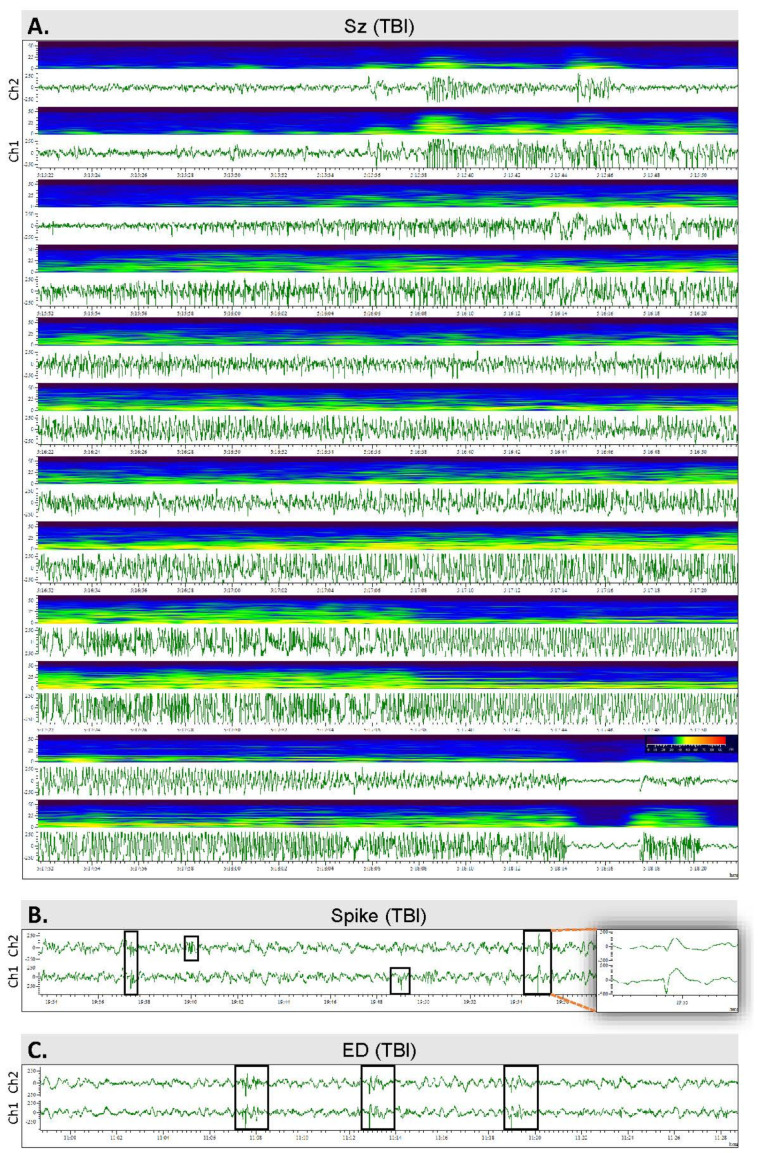
Spontaneous epileptiform activity: (**A**) An unprovoked spontaneous seizure in a TBI rat #36. The seizure started at the N3–REM transition and lasted for 166 s. (**B**) Epileptiform spikes in a TBI rat #36. The insert on the right shows the spike with a higher resolution. (**C**) Epileptiform discharges (EDs) in a TBI rat (#36).

On D180, rats were reconnected to the vEEG for 30 days to monitor the occurrence of unprovoked epileptiform activity (Figure 4). Seizures were detected from vEEG recordings by browsing the files visually on a computer screen. Rats with at least 1 seizure were diagnosed with PTE [27]. In addition, the presence of EDs and spikes was noted.

### 2.8. Scoring of Sleep

**Figure 5:**
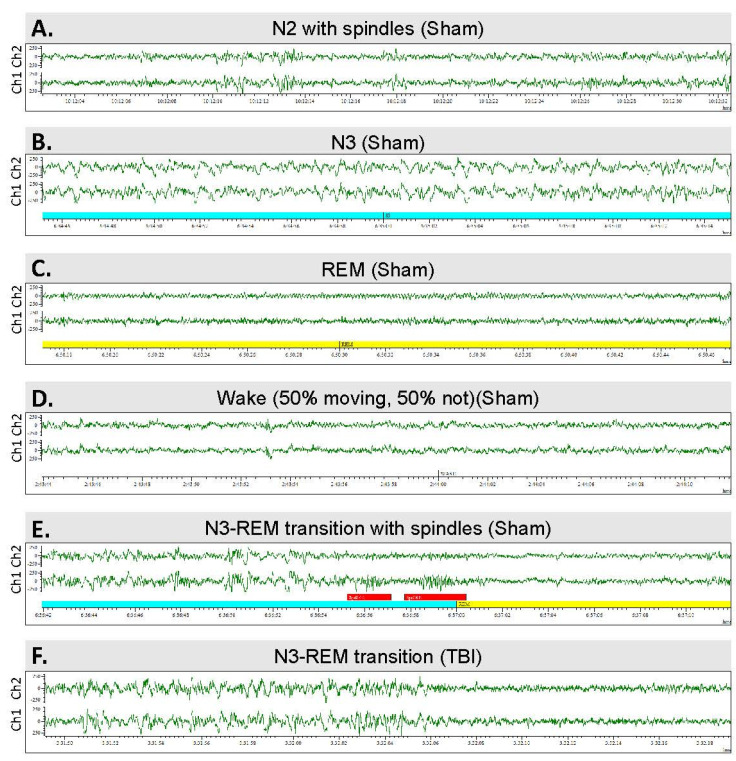
Sleep architecture—the sleep–wake stages analyzed: (**A**) Stage N2 sleep with spindles. (**B**) Stage N3. (**C**) Rapid eye movement (REM) sleep. (**D**) Waking EEG. (**E**) N3–REM transition sleep period in a sham-operated rat. Note the K-complexes and sleep spindles (red bars). (**F**) N3–REM transition sleep period in a rat with traumatic brain injury and post-traumatic epilepsy.

A 24-h epoch on the 15th day of a 30-day recording during the 7th post-TBI month was analyzed. In case of the occurrence of seizures or a poor-quality EEG recording, a 24-h EEG epoch starting 48 h earlier was included in the analysis. Sleep was scored as recommended by the American Academy of Sleep Medicine, with slight modifications [28]. Briefly, the 24-h EEG was divided into 30-s epochs and scored as awake (W), stage II (N2), stage III (N3), or REM sleep (Figure 5). The score annotated to each individual epoch was based on visual review and identification of the specific characteristics dominating over 50% of the duration of a given 30-s epoch. Briefly, N2 is characterized by spindles and K complexes, N3 by generalized high-amplitude activity at the delta frequency, and REM sleep by generalized low-amplitude theta activity. The scoring permitted quantification and comparison of the quality (sleep fragmentation) and duration of sleep variables. First, sleep patterns were analyzed separately during the dark and light periods. A hypnogram was generated (homemade script, Spike v. 10.09) to visualize sleep over the circadian rhythm. A sleep fragmentation index (SFI), modified for use in rodents, was calculated [29]. First, the number of transitions from a deeper to a lighter stage of sleep, or to wakefulness, were counted (lights-on, lights-off, and both combined). Then, the total number of transitions from any sleep stage to a different stage of sleep or wakefulness was counted (lights-on, lights-off, and both combined). Finally, the number of transitions was divided by the recording time, in hours (lights-on, lights-off, and both combined).

### 2.9. Histology

To assess the location and extent of the FPI and exclude non-TBI-related epileptogenic lesions (e.g., abscesses), rats were perfused for histology after completing the vEEG (D210).

Perfusion: Briefly, rats were deeply anesthetized with an intraperitoneal injection of pentobarbital (60 mg/kg), and perfused transcardially via the aorta with 0.9% NaCl (6 min, 30 mL/min), followed by 4% paraformaldehyde in 0.1 M sodium phosphate buffer, pH 7.4 (30 min, 30 mL/min). The brain was removed from the skull, fixed in 4% paraformaldehyde for 4 h, cryoprotected in 20% glycerol in 0.02 M potassium phosphate-buffered saline (pH 7.4) for 24 h, frozen in dry ice, and stored at −70 °C for further processing.

Frozen coronal sections of the brain were cut (30 µm thick, 1-in-5 series) using a sliding microtome. The first series of sections was stored in 10% formalin at room temperature and used for thionin staining. Other series of sections were collected into tissue collection solution (30% ethylene glycol, 25% glycerol in 0.05 M sodium phosphate buffer) and stored at −20 °C until processed.

Nissl staining: The first series of sections was stained with thionin, cleared in xylene, and coverslipped using DePeX^®^ (BDH Chemical, Poole, UK) as a mounting medium.

Preparation of unfolded cortical maps: To assess the cortical lesion area and damage to different cytoarchitectonic cortical areas after TBI, all thionin-stained sections were digitized (panoramic images of the coronal sections were captured with a Zeiss Imager 2 microscope). Unfolded cortical maps were then prepared from the digitized histologic sections as previously described in [30], by applying in-house software (https://unfoldedmap.org, accessed on 16 April 2022) that was adapted to the rat brain [31].

### 2.10. Statistics

Statistical analyses were performed using SPSS for Windows (v. 27.0). Treatment and time effects on body weight, composite neuroscore, and the PTZ seizure-susceptibility test were analyzed using repeated-measures analysis of variance (ANOVA), followed by post hoc Bonferroni correction for multiple tests. Within each treatment group, the evolution of changes was assessed using Friedman’s 2-way ANOVA or a related-samples Cochran’s Q test, followed by post hoc Bonferroni correction for multiple tests. Intra-timepoint comparisons between the animal groups were tested with the nonparametric Kruskal–Wallis test, followed by post hoc analysis with the Mann–Whitney U test (i.e., body weight, composite neuroscore, cortical area, sleep parameters, PTZ seizure susceptibility test). Cohen’s delta was assessed as an indicator of effect size. The sensitivity and specificity of the single PTZ test and sleep parameters were assessed by receiver operating characteristic (ROC) analysis. To assess the presence of model overfitting, the logistic regression fitting and ROC analysis were repeated using a leave-one-out cross-validation, implemented in MATLAB (MATLAB and Statistics and Machine Learning Toolbox Release 2018b; The MathWorks, Inc., Natick, MA, USA). The cross-validated area under the ROC curve (AUC) was estimated using the pooling method [32] and Brian Lau’s MATLAB AUC codes (https://github.com/brian-lau/MatlabAUC, accessed on 16 April 2022). The 95% confidence interval for the cross-validated AUC was estimated using a bias-corrected and accelerated bootstrap method. In all tests of statistical significance, a *p* value < 0.05 was considered significant. Data are shown as the mean ± standard deviation of the mean (unless otherwise specified).

## 3. Results

### 3.1. Mortality and Exclusions

Acute (<48 h) post-impact mortality was 15% (5/34) in the TBI group and 0% (0/16) in the sham-operated group. Follow-up mortality due to unknown causes was 3% (1/29). Thus, 16 sham-operated and 28 TBI rats underwent the PTZ seizure-susceptibility test, followed by 1 month of continuous vEEG monitoring (Figure 2).

### 3.2. Body Weight

**Figure 6:**
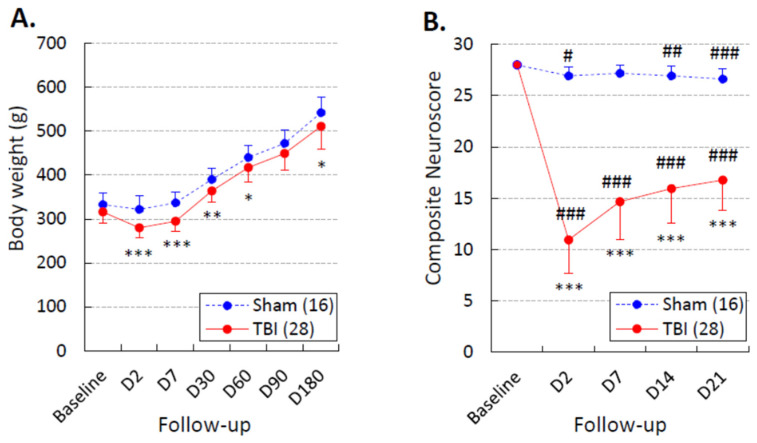
Body weight and performance in the composite neuroscore test: (**A**) Body weight was slightly lower in the traumatic brain injury (TBI) group (red line) than in the sham group (dashed blue line) over the 180-day monitoring period (repeated-measures ANOVA: time effect *p* < 0.001, group effect *p* < 0.01, time x group effect *p* > 0.05). (**B**) The composite neuroscore test was performed before the first pentylenetetrazol (PTZ) test session. Although rats with TBI showed somatomotor recovery between D2 and D7 (*p* < 0.001, Wilcoxon), they did not reach the performance level of sham-operated experimental controls during the 3-week post-injury period (repeated-measures ANOVA: time effect *p* < 0.001, group effect *p* < 0.001, time x group effect *p* < 0.001). Data are shown as the mean ± standard deviation of the mean. Statistical significance: *, *p* < 0.05; **, *p* < 0.01; ***, *p* < 0.001 (Mann–Whitney U test compared with the sham group); #, *p* < 0.05; ##, *p* < 0.01; ###, *p* < 0.001 (Wilcoxon test compared with the baseline).

Changes in body weight over the 180-day follow-up are summarized in Figure 6A. On D2, the body weight was 97% that of baseline in the sham group (322 ± 31 vs. 333 ± 28 g, *p* > 0.05) and 89% in the TBI group (280 ± 22 vs. 316 ± 24 g, *p* > 0.05). Over the follow-up, the body weight remained slightly lower in the TBI group than in the sham group (*p* < 0.001). In both groups, the body weight increased after the first week compared with that on D2 (Figure 6A).

### 3.3. Composite Neuroscore

The severity of the post-TBI somatomotor impairment and recovery over the 21-day follow-up is summarized in Figure 6B. On D2, the average neuroscore was 26.9 ± 0.9 in the sham group and 10.8 ± 3.2 in the TBI group (*p* < 0.001). Although rats with TBI showed somatomotor recovery from D2 to D7 (*p* < 0.001), they did not reach the performance levels of sham-operated experimental controls during the 3-week post-injury follow-up.

### 3.4. Spontaneous Epileptiform Activity

Monitoring: Forty-four rats (16 sham, 28 TBI) completed the PTZ test follow-up and 1-month video EEG monitoring (Figure 2). Of the 28 rats with TBI, 6 were excluded from the analysis of electrographic seizures and other epileptiform activities due to an insufficient EEG quality.

Occurrence of epileptiform activity: Altogether, 15 of the remaining 22 TBI rats showed epileptiform activity in EEG (TBIe+ group) and 7 did not (TBIe−) (Figure 2), including seizures, periodic EDs, periodic sharp delta activity, or spikes within the N3 or N2 sleep spindles (Figure 4B,C).

We also observed some epileptiform activity in 3 of the 16 sham-operated animals during the 7th month after the sham operation (craniectomy), including spikes within N3 spindles, periodic EDs, and trains of large-amplitude sharp waves.

Occurrence of unprovoked seizures: Four of the twenty-two TBI rats had at least one unprovoked electrographic seizure over the 4-week monitoring during the 7th month post-TBI (TBI+), and eighteen did not (TBI−). That is, 18% (4/22) of the monitored TBI rats had developed PTE (Figure 2 and Figure 4A). Rat #4 had a 29-s seizure during the N2 sleep stage. Rat #7 had a 22-s seizure during an awake period. Rat #22 had 2 seizures: a 38-s seizure, and a 28-s seizure 4 days later, both at the N3–REM transition. Rat #36 had 6 seizures with an average duration of 105 ± 33 s, ranging from 60 to 150 s; 4 of the seizures occurred in a cluster (i.e., >3 seizures/24 h).

### 3.5. Evolution of Seizure Susceptibility in the PTZ Test

**Figure 7:**
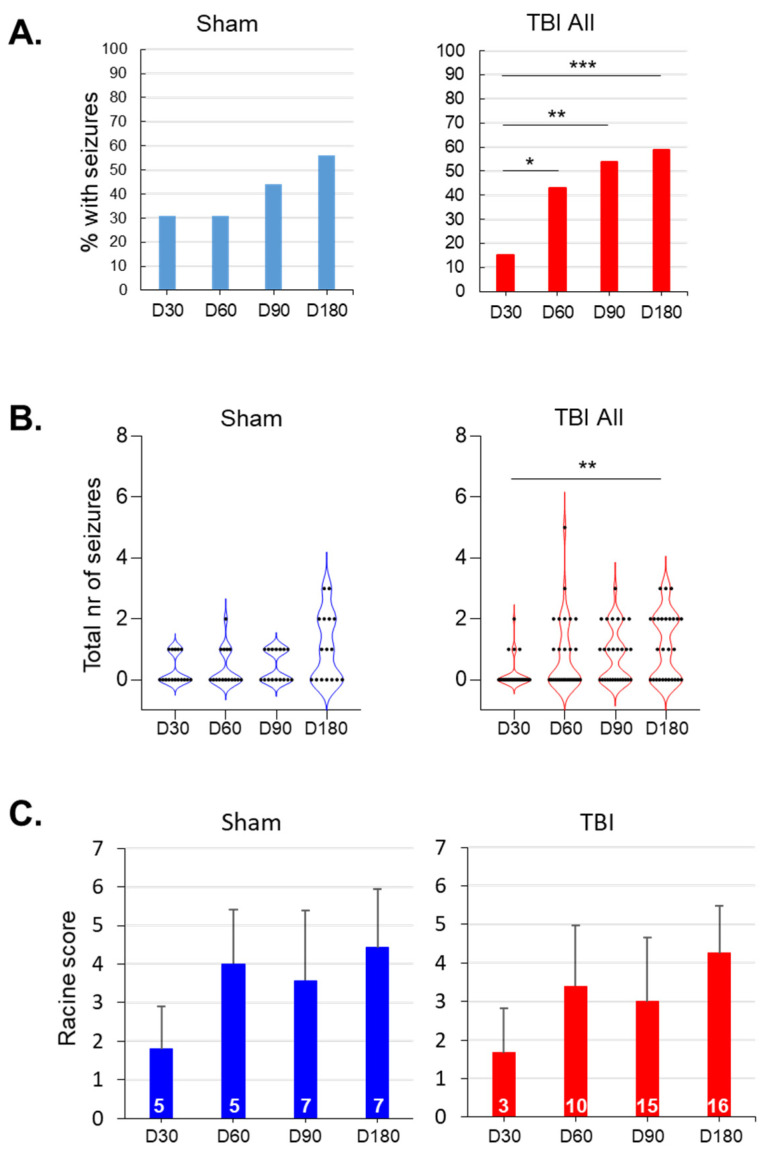
Evolution of seizure susceptibility over the 7-month follow-up: (**A**) Bar graphs showing the percentage of rats (*y*-axis) with pentylenetetrazol (PTZ)-induced seizures on day (D) 30, D60, D90, and D180 after sham operation (*n* = 16) or traumatic brain injury (TBI; *n* = 28). In the TBI group, the percentage of rats with seizures increased over time (adjusted *** *p* < 0.001, ** *p* < 0.01, * *p* < 0.05 compared with that at D30; related-samples Cochran’s Q test). (**B**) Violin plots showing the total number of PTZ-induced seizures (*y*-axis) in each animal during different testing sessions. Each black dot represents 1 animal. The general linear model revealed a significant effect of time on the seizure count (*p* < 0.001), but no group or time x group effects (*p* > 0.05). Although the seizure number increased in both the sham-operated and TBI animals, the only significant difference detected was in the TBI group when the D30 and D180 timepoints were compared (adjusted ** *p* < 0.01; related-samples Friedman’s 2-way ANOVA). (**C**) Average Racine score (± standard deviation) of the first PTZ-induced seizure on each testing day (*y*-axis). The general linear model revealed a significant effect of time on the seizure count (*p* < 0.001), but no group or time x group effects (both *p* > 0.05). In the TBI group, the Racine score tended to increase over time (related-samples Friedman’s 2-way ANOVA, *p* < 0.05), but pairwise comparisons did not survive Bonferroni corrections for multiple testing (adjusted *p* > 0.05). The numbers within each bar indicate the number of rats with seizures on that testing day.

EEG quality was sufficient for analysis of induced and unprovoked epileptiform activity in all 16 sham cases and 28 TBI cases. The PTZ-induced seizure-susceptibility test data are summarized in Figure 3D–F and Figure 7, Table 1, and Supplementary Tables S1, S2, and S7.

#### 3.5.1. Sham vs. TBI

The Mann–Whitney U test revealed no differences between the TBI and sham groups in any of the PTZ test parameters on D30, D60, D90, or D180 (Table 1). However, as the variability between the animals was large, we assessed Cohen’s delta to estimate the effect size (Table 1, Supplementary Tables S1 and S2). Because a moderate-to-large effect size was observed in several parameters, suggesting increased seizure susceptibility in the TBI group (Table 1), we next assessed the dynamics of seizure susceptibility in the sham and TBI groups separately.

Sham-operated experimental controls: We observed no increase in seizure susceptibility over time in the sham group (Table 1).

TBI group—all animals: The percentage of rats with PTZ-induced seizures increased over time compared with that at D30 (*p* < 0.001, related-samples Cochran’s Q test) (Figure 7A). The number of seizures after PTZ injection also increased from 0.19 ± 0.48 on D30 to 1.15 on D180 (adjusted *p* < 0.01, Friedman’s 2-way ANOVA with Bonferroni correction) (Figure 7B; Table 1). The latency to the first ED decreased from 480 ± 662 s on D30 to 176 ± 138 s on D90 (adjusted *p* < 0.05, Friedman’s 2-way ANOVA with Bonferroni correction) (Table 1). The latency to the first seizure decreased from 370 ± 209 s on D30 to 177 ± 93 s on D90 (adjusted *p* < 0.05, Friedman’s 2-way ANOVA with Bonferroni correction) (Table 1).

#### 3.5.2. TBIe− vs. TBIe+

The latency to the first PTZ-induced ED was shorter in the TBIe+ group than in the TBIe− group on D90 (164 ± 180 s vs. 207 ± 71 s, *p* < 0.05) and on D180 (194 ± 215 s vs. 316 ± 114 s, *p* < 0.01) (Supplementary Table S1).

In the TBIe− group (*n* = 7), the latency to the first ED decreased from 976 ± 833 s on D30 to 207 ± 71 s on D90 (adjusted *p* < 0.05, Friedman’s 2-way ANOVA with Bonferroni correction) (Supplementary Table S1).

In the TBIe+ group (*n* = 7), latency to the PTZ-induced first seizure decreased from 370 ± 209 s on D30 to 144 ± 87 s on D90 (adjusted *p* < 0.05, Friedman’s 2-way ANOVA with Bonferroni correction) (Supplementary Table S1).

#### 3.5.3. TBI+ vs. TBI−

The TBI+ group contained only four animals (see below). No differences were detected between the TBI+ and TBI−groups at any of the testing points (Supplementary Table S2). However, a trend toward an increased number of PTZ-induced seizures was observed in the TBI+ group over the course of the 7-month follow-up (adjusted *p* = 0.061, Friedman’s 2-way ANOVA with Bonferroni correction; Supplementary Table S2).

### 3.6. Sleep

A total of 5 animals in the sham group and 14 in the TBI group were excluded from the sleep analysis due to noisy EEG channels. Thus, sleep EEG was analyzed in 11 sham-operated and 14 TBI rats (Figure 2). The average duration of sleep recordings was 24.14 ± 0.14 h in the sham group and 24.05 ± 0.06 h in the TBI group (*p* > 0.05). Data are summarized in Table 2, Table 3 and Table 4 and Supplementary Tables S3–S6.

#### 3.6.1. Sham vs. TBI

Total duration of sleep: Both the sham-operated controls and TBI rats slept (duration of N2, N3, and REM combined) for approximately 60% of the 24-h recording time (Table 2). No differences were detected in the total duration of sleep (sham 14.62 ± 1.50 h vs. TBI 14.14 ± 1.29 h) or waking periods (sham 9.34 ± 1.53 h vs. TBI 9.85 ± 1.11) between groups (all *p* > 0.05). As expected, the total duration of sleep was shorter during the lights-off period than during the lights-on period (12 h/12 h) in both the sham group (*n* = 11, lights-off 5.58 ± 0.89 h vs. lights-on 9.04 ± 0.71 h, *p* < 0.01) and the TBI group (*n* = 14, lights-off 5.14 ± 0.57 h vs. lights-on 9.00 ± 0.78 h, *p* < 0.01). The duration of sleep was comparable between the sham and TBI groups during the lights-off period (sham 5.58 h ± 0.89 h vs. TBI 5.14 ± 0.57 h) and the lights-on period (sham 9.04 ± 0.71 h vs. TBI 9.00 ± 0.78 h) (Table 2).

Duration of different sleep–awake stages: Next, we assessed the duration of different sleep–awake stages during the lights-off and lights-on periods (Table 3). During the lights-off period, the average duration of REM was approximately 16% shorter in the TBI group than in the sham group (1.61 ± 0.05 min vs. 1.91 ± 0.09 min, *p* < 0.01). Moreover, when the lights-on and lights-off periods were combined, the average duration of REM periods was shorter in the TBI group than in the sham-operated rats (12%, 1.81 ± 0.15 min vs. 2.04 ± 0.25 min, *p* < 0.05).

Transitions and sleep fragmentation: During the lights-on period, the number of transitions to N3 sleep was greater in the TBI group than in the sham group (TBI 86.50 ± 4.14 vs. sham 73.45 ± 2.66, *p* < 0.05). Moreover, the number of transitions to the REM stage was greater in the TBI group than in the sham group (TBI 70.93 ± 4.7 vs. sham 59.73 ± 3.28, *p* < 0.05). Overall, the total number of transitions during the lights-on period was greater in the TBI group than in the sham group (TBI 226.21 ± 10.91 vs. sham 184.73 ± 9.88, *p* < 0.05, Table 4). Furthermore, when the lights-on and lights-off periods were combined, the total number of transitions was approximately 15% higher in the TBI group than in the sham group (TBI 396.93 ± 13.70 vs. sham 346.09 ± 18.39, *p* < 0.05, Table 4). Consequently, sleep fragmentation was greater in the TBI group than in the sham-operated animals during the lights-on period (TBI 18.67 ± 0.94 vs. sham 15.31 ± 0.81, *p* < 0.05), and when the lights-on and lights-off periods were combined (TBI 16.46 ± 0.59 vs. sham 14.40 ± 0.94, *p* < 0.05) (Table 4).

**Figure 8:**
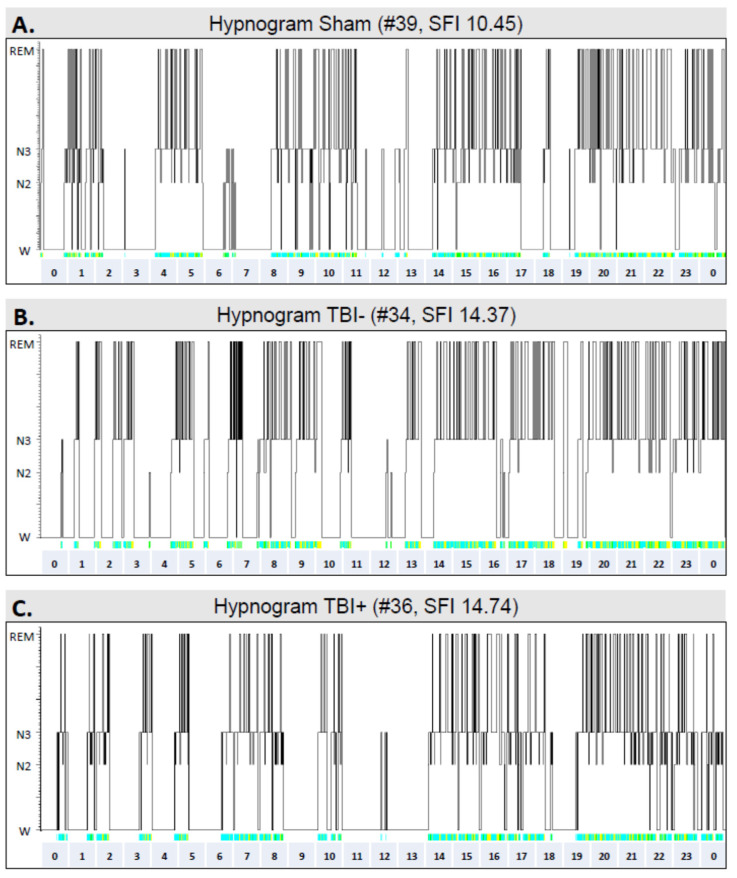
Hypnograms showing the sleep architecture in different groups over a 24-h period in the 7th follow-up month: (**A**) A sham-operated control (#39). (**B**) A rat (#34) with TBI but no epilepsy (TBI−). (**C**) A rat (#36) with TBI and epilepsy (TBI+). Note the fragmented sleep–awake cycle in TBI animals. Abbreviations: N2, N2 sleep stage; N3, N3 sleep stage; REM, rapid eye movement sleep; SFI, sleep fragmentation index; W, awake.

Figure 8 shows representative hypnograms from a sham animal (rat #39, combined fragmentation index of the lights-on and lights-off periods: 10.45) as well as from two rats with TBI—one without epilepsy (rat #34, fragmentation index: 14.37) and the other with epilepsy (rat #36, fragmentation index: 14.74).

#### 3.6.2. TBIe+ vs. TBIe−

Total duration of sleep: The duration of awake and sleep periods during the lights-on or lights-off periods did not differ between the TBIe+ and TBIe−groups (Table 2).

Duration of different sleep–awake stages: The average duration of N2 periods was shorter in the TBIe+ group than in the TBIe− group during the lights-off period (1.12 ± 0.17 min vs. 1.43 ± 0.30 min, *p* < 0.05), as well as when the lights-on and lights-off periods were combined (1.30 ± 0.19 min vs. 1.63 ± 0.24 min, *p* < 0.05) (Supplementary Table S3). The average duration of N3 periods was longer in the TBIe+ group than in the TBIe− group (3.63 ± 0.51 min vs. 2.99 ± 0.48 min, *p* < 0.05). The duration of REM sleep was shorter in the TBIe+ group than in the TBIe− group during the lights-on period (2.06 ± 0.46 h vs. 2.92 ± 0.72 h, *p* < 0.05).

Transitions and sleep fragmentation: The number of transitions from N3 to the awake stage was greater in the TBIe+ group than in the TBIe− group during the lights-on period (11.14 ± 5.00 vs. 5.00 ± 1.13, *p* < 0.05), during the lights-off period (12.86 ± 1.56 vs. 8.14 ± 3.00, *p* < 0.05), and when the lights-on and lights-off periods were combined (24.00 ± 2.86 vs. 13.14 ± 2.41, *p* < 0.05) (Supplementary Table S4).

#### 3.6.3. TBI+ vs. TBI−

Total duration of sleep: The duration of the awake and sleep periods during the lights-on and lights-off periods did not differ between the TBI+ and TBI− groups (Table 2).

Duration of different sleep–awake stages. The average duration of N3 periods was longer in the TBI+ group than in the TBI− group during the lights-off period (3.86 ± 0.73 min vs. 2.86 ± 0.36 min, *p* < 0.05) (Supplementary Table S5).

Transitions and sleep fragmentation: The TBI+ rats had a substantially greater number of transitions from N3 to the awake stage than the TBI−rats during the lights-on period (15.00 ± 3.46 vs. 6.18 ± 4.06, *p* < 0.05), as well as when the lights-on and lights-off periods were combined (28.67 ± 4.06 vs. 15.82 ± 2.16, *p* < 0.05) (Supplementary Table S6).

### 3.7. Occurrence of SWDs

In the same 24-h epoch that was used for the sleep disturbance analysis, we also analyzed the occurrence of spike-and-wave discharges (SWDs), which are described as normal oscillations in many rat strains [33].

**Figure 9:**
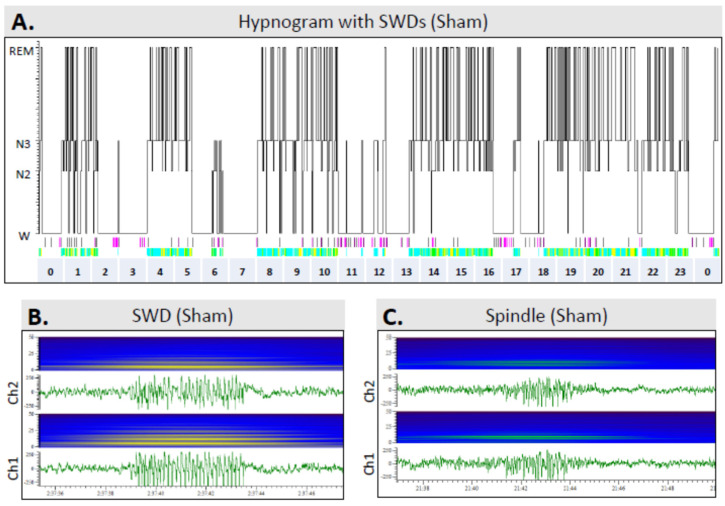
Occurrence of spike-and-wave discharges (SWDs) over the 24-h sleep–awake cycle: (**A**) A hypnogram in a sham-operated experimental control rat (#39). (**B**) An SWD and associated power spectrum in a sham-operated rat (#39). SWDs are indicated by light-green bars below the hypnogram (panel (**A**)). Note their occurrence only during awake periods. Moreover, note the harmonics in the power spectrum. (**C**) An N3 sleep spindle in a sham-operated rat (#39), and corresponding power spectrum. Occurrence of sleep spindles is indicated by purple bars below the hypnogram in panel (**A**). Note the single frequency band in the power spectrum. Abbreviations: Ch, electroencephalogram recording channel (see Figure 3B); N2, N2 sleep stage; N3, N3 sleep stage; REM, rapid eye movement sleep; W, awake.

Sham vs. TBI: Of the 11 rats in the sham group and 14 in the TBI group, 8 (73%) and 9 (64%), respectively, showed SWDs (*p* > 0.05) in EEGs recorded during the 7th post-injury month. On average, the number of SWDs per 24-h EEG epoch was 127 ± 96 (*n* = 11, median 144, range 0–273) in the sham rats and 127 ± 133 (*n* = 14, median 107, range 0–386) in the TBI group (*p* > 0.05). In both groups, SWDs occurred exclusively during the awake stage (Figure 9).

TBIe+ vs. TBIe−: The number of SWDs per 24 h was 63 ± 87 in rats without epileptiform activity (*n* = 7, median 46, range 0–206) and 192 ± 145 in rats with epileptiform activity (*n* = 7, median 179, range 0–386; *p* > 0.05).

TBI+ vs. TBI−: The number of SWDs per 24 h was 76 ± 81 in rats without epilepsy (*n* = 11, median 77, range 0–206) and 316 ± 117 in rats with epilepsy (*n* = 3, median 382, range 181–386; *p* < 0.05).

### 3.8. Cortical Lesion Area

Analysis of unfolded cortical maps drawn from histologic sections prepared 7 months post-TBI revealed the lesion core in the auditory association cortex (AuV, Au1, AuD) (Figure 3C). The mean cortical lesion area was 19.78 ± 14.85 mm^2^ (range 5.61–76.38 mm^2^).

Rats with PTZ-induced seizures at any timepoint had a smaller cortical lesion area than rats without PTZ-induced seizures (14.70 ± 6.86 vs. 27.24 ± 19.56 mm^2^, *p* < 0.01; Cohen’s d 1.02). No difference was detected between TBI rats with or without epilepsy (i.e., with or without unprovoked seizures; 18.80 ± 12.10 vs. 20.00 ± 15.69 mm^2^, *p* > 0.05; Cohen’s d 0.08) or epileptiform activity (17.42 ± 9.30 vs. 24.82 ± 22.93 mm^2^, *p* < 0.01; Cohen’s d 0.50).

### 3.9. PTZ Test Parameters as Biomarkers of TBI and Epileptogenesis

Next, we assessed each PTZ test parameter as a diagnostic biomarker of TBI and epileptogenesis (Table 5, Supplementary Table S7).

#### 3.9.1. PTZ Test Parameters as Diagnostic Biomarkers of TBI

None of the PTZ test parameters alone was able to differentiate TBI rats from sham-operated controls (all AUCs *p* > 0.05, Supplementary Table S7).

A combination of three PTZ test parameters—including the latency to the first ED, number of EDs, and number of PTZ-induced seizures on D60—resulted in an AUC of 0.679 (*p* = 0.05), and survived the leave-one-out cross-validation with a misclassification rate of 36% (Table 5). The best differentiation was achieved when the above three parameters were combined with latency to the first PTZ-induced seizure and duration of the first PTZ-induced seizure on D90 (cross-validated AUC 0.733, *p* < 0.05; misclassification rate 32%) (Table 5).

#### 3.9.2. PTZ Test Parameters as Diagnostic Biomarkers of Epileptogenesis

##### TBIe+ vs. TBIe−

Some of the individual parameters—particularly latency to the first ED and latency to the first PTZ-induced seizure—showed promise as biomarkers of epileptogenesis, differentiating the TBIe+ rats from the TBIe− rats (Supplementary Table S7).

A combinatory biomarker approach revealed that a combination of three parameters—including latency to the first ED, number of EDs, and number of PTZ-induced seizures on D90—resulted in an AUC of 0.743 (*p* = 0.05), and survived the leave-one-out cross-validation with a misclassification rate of 36%. On D180, the AUC was 0.752 (*p* < 0.05), with a misclassification rate of 32% (Table 5).

##### TBI+ vs. TBI−

Some of the PTZ parameters showed an AUC of ~0.800 in the ROC analysis. However, only three animals were in the TBI+ group; therefore, the data should be considered preliminary (Supplementary Table S8).

None of the combinatory biomarkers survived the leave-one-out cross-validation (Table 4).

### 3.10. Sleep Disturbance Parameters as Biomarkers of TBI and Epileptogenesis

Data are summarized in Table 5 and Supplementary Table S8.

#### 3.10.1. Sleep Disturbance Parameters as Diagnostic Biomarkers of TBI 

Several individual sleep parameters assessed in the 7th post-TBI month—including average duration of REM periods during the lights-off period or the lights-on and lights-off periods combined, number of transitions to N3 during lights-on, number of transitions to REM during lights-on, total number of transitions when the lights-on and lights-off periods were combined, fragmentation index during lights-on, and fragmentation index when the lights-on and lights-off periods were combined—had an AUC > 0.740 (*p* < 0.05), thus showing promise as diagnostic biomarkers of prior TBI (Supplementary Table S8).

A combination of four indicators of sleep disturbance (average duration of REM episodes (lights-off), number of transitions to N3 (lights-on), total number of transitions (lights-on), and fragmentation index (lights-on)) resulted in an AUC of 0.792, and survived the leave-one-out validation with a misclassification rate of 28% as a diagnostic biomarker of prior TBI (Table 5).

#### 3.10.2. Sleep Disturbance Parameters as Diagnostic Biomarkers of Epileptogenesis

##### TBIe+ vs. TBIe−

Several individual sleep disturbance parameters showed potential as diagnostic biomarkers for differentiating rats with and without epileptiform activity in EEG (Supplementary Table S8). In particular, the number of transitions from N3 to the awake stage had an AUC of 0.827 during the lights-on period (95%CI 0.598–1.055; *p* < 0.01) and an AUC of 0.847 during the lights-off period (95%CI 0.631–1.063; *p* < 0.01) (Supplementary Table S8).

Although several combinations of sleep disturbance parameters showed promise as biomarkers for differentiating the TBIe+ and TBIe- groups, none of them survived the leave-one-out validation. Adding PTZ parameters did not improve the combinatory sleep biomarkers’ performance (Table 5).

##### TBI+ vs. TBI−

Several individual sleep disturbance parameters showed potential as diagnostic biomarkers for differentiating rats with and without epilepsy (Supplementary Table S8). However, the TBI+ group included only three animals; therefore, the data should be considered preliminary—particularly as the combinatory biomarkers, which comprised several sleep disturbance parameters, did not survive the leave-one-out validation (Table 5).

## 4. Discussion

The present study investigated whether seizure susceptibility increases over a period of weeks to months after TBI, and whether seizure susceptibility predicts the development of PTE in rats. Moreover, we examined whether TBI induces sleep disturbances—particularly in the N3 and REM periods, which are the most susceptible periods for seizure occurrence. Finally, we evaluated whether the PTZ test and/or the sleep parameters could provide biomarkers for prior TBI and ongoing epileptogenesis.

### 4.1. Rats with TBI Showed A Mild Progression in Seizure Susceptibility over the 6-Month Follow-Up

Our previous studies in eight independent rat cohorts consistently demonstrated increased seizure susceptibility in the PTZ test at 3.5, 4, 6, 11, or 12 months after lateral FPI [11,34,35,36,37,38,39,40]. Other investigators also found increased seizure susceptibility caused by PTZ in rats at 4 days [21], 2 weeks [41], 5 weeks [42,43], 6 weeks [44,45,46], and 12 weeks [47] after lateral FPI (literature review in Supplementary Table S9). Mice with lateral FPI also exhibit increased excitability when tested at 30 days or 12 months after lateral FPI [48,49]. Seizure susceptibility in the PTZ test has been reported after controlled cortical impact (CCI) [48] and weight-drop-induced TBI [50]. Thus, even though the parameters assessed as indicators of seizure susceptibility and the types of monitoring differ between laboratories (observation vs. video vs. EEG vs. vEEG) (Supplementary Table S9), a single administration of PTZ seems to consistently confer increased seizure susceptibility after lateral FPI.

In the present study, we administered PTZ four times. On the first testing day (D30), 30% of the rats in the sham group and 15% in the TBI group exhibited PTZ-induced seizures. We considered this acceptable for monitoring the later anticipated progression in seizure incidence. In fact, in the TBI group, the percentage of rats with PTZ-induced seizures was increased on the later testing days compared with that at D30. However, we found no progressively increasing differences between the sham and TBI groups in the average latency to the first spike, ED, or seizure, nor did the number of spikes, EDs, or PTZ-induced seizures, or the duration of the first seizure, differ between the groups on any testing day. This may relate to a large variability in seizure susceptibility between the rats at a given timepoint, as previously reported in [45], showing great variability in the PTZ dose needed to induce convulsive seizures at 6 weeks post-TBI. Moreover, our recent functional magnetic resonance imaging study revealed that the outcome parameters used in the PTZ test are affected by the onset pattern of the PTZ-induced seizures. For example, the latency to the blood-oxygen-level-dependent activation signal was slower in rats with a focal perilesional seizure onset than in those with bilateral generalized seizure onset [35]. However, Cohen’s delta revealed a medium-to-large effect size, which was most clear on D90, indicating a trend towards reduced latency to the first ED and the first seizure in the TBI group compared with the sham group. The effect size was also medium–large when the latency to the first seizure was compared between the TBI and sham groups on D30 and D180, or when the duration of the first seizure was compared between groups on D30 and D180, suggesting greater seizure susceptibility in the TBI group. Interestingly, group-specific analysis indicated progression of seizure susceptibility in the TBI group only. The most remarkable increase was found from D30 to D90 or to D180, when the latency to the first ED or the first seizure, or the average number of seizures, was used as the test parameter. Interestingly, Saraiva et al. [21] administered PTZ to rats with severe lateral FPI twice, on D4 and D8 post-injury, and found increased seizure susceptibility on D4, but not on D8. Taken together, although seizure susceptibility increased after lateral FPI, the progression was not linear, and it was not observed in all of the PTZ test parameters assessed.

In 18% of the vEEG-monitored TBI animals, unprovoked seizures were observed during the 7th post-TBI month—slightly less than the expected 25% [23,51]. Unlike the SWDs, which occurred during the waking period, unprovoked seizures typically occurred during sleep, as described previously [52]. Although the comparisons between the TBI+ and TBI− groups should be interpreted cautiously due to the low animal numbers, Cohen’s delta indicated a moderate-to-large effect size between the TBI+ and TBI− groups in the latency to the first spike, ED, or seizure, as well as in the numbers of spikes, EDs and seizures, suggesting increased seizure susceptibility in the TBI+ group compared with the TBI− group. The increased seizure susceptibility was even more evident when rats exhibiting any epileptiform activity were compared with TBI rats exhibiting no epileptiform activity. We also found some epileptiform activity in 19% of sham-operated experimental controls. Whether this was related to the mild cortical inflammation associated with craniectomy remains to be studied [53].

### 4.2. TBI Causes Chronic Sleep Disturbances

We used the entire 24-h epoch sampled during the 7th post-injury month to analyze sleep disturbances in rats with TBI. The EEG epoch was carefully chosen from the recording period without preceding seizure occurrence. To our best knowledge, this is the longest epoch of EEG analyzed for sleep in rats, and the first study in animals with post-traumatic epileptiform activity.

TBI induces chronic sleep disturbances in humans with TBIs, and in several animal models of TBI, including lateral FPI [51,52,53,54,55,56,57,58,59,60,61,62,63]. Our data extend previous findings by showing reduced REM sleep in injured animals. The transitions to N3 and REM sleep were more frequent; consequently, the TBI group experienced more fragmented sleep than the sham group.

As unprovoked seizures in rats with PTE almost exclusively occur during the N3–REM transition [58], we hypothesized that rats with seizures and/or epileptiform activity would have experience greater sleep disturbance than injured rats without epileptiform activity. In the present cohort, four rats showed unprovoked late seizures. Consistent with our previous findings, most of the seizures occurred at the N3–REM transition; that is, instead of entering REM, the animals developed a seizure, which was typically followed by arousal within a few seconds after seizure onset (“woke-up”). Another three rats also showed sleep-related epileptiform activity in EEG—including spikes within K-complexes and spindles—during REM sleep. We observed no consistent differences in the average duration of the N3 or REM sleep periods, number of transitions, or fragmentation index between the injured rats with or without epileptiform activity, or with or without PTE, which was probably related to the small animal numbers in these subgroups. However, the rats with epileptiform activity or PTE showed a 1.8-fold increase in the number of transitions from N3 to awake during a 24-h epoch, indicating difficulty entering the REM phase and maintaining sleep.

### 4.3. PTZ Test and Sleep Disturbance Parameters as Biomarkers of TBI and Epileptogenesis

Our study showed that a combination of three PTZ test parameters—including the latency to the first ED, number of EDs, and number of PTZ-induced seizures—formed a sensitive and specific diagnostic biomarker of the prior occurrence of TBI. Starting on D90, the combinatory biomarkers were also able to differentiate the rats with and without epileptiform activity, with an AUC of approximately 0.750, and with 77–79% accuracy. All of these parameters can be easily measured in EEG recordings, and are therefore expected to be reproducible in different laboratories.

Several sleep parameters reporting on chronic sleep disturbances also demonstrated moderate sensitivity as biomarkers of the occurrence of prior TBI. The best biomarker performance was achieved by combining the average duration of REM sleep periods, number of transitions to N3, total number of transitions, and the fragmentation index, resulting in a moderate AUC of 0.792 and 77% accuracy.

Like the individual PTZ test parameters, several individual sleep disturbance parameters (e.g., number of transitions from N3 to awake) also differentiated rats with or without epileptiform activity or unprovoked seizures, with an AUC > 0.800. However, considering the small sample size, the data obtained should be considered preliminary. Moreover, the combinatory sleep disturbance biomarker approach did not reveal any biomarkers that survived the leave-one-out validation. In contrast to our expectations, biomarker performance was not improved by combining the PTZ test and sleep disturbance parameters.

Overall, TBI animals show increased seizure susceptibility and sleep disturbance, which are further worsened in animals with epileptiform activity. The promising data on their potential as biomarkers for epileptogenesis warrant validation in a larger, independent animal cohort.

### 4.4. Methodological Considerations

The major difference between the present and previous studies in the lateral FPI model was that we assessed PTZ-induced excitability several times over the period of 6 months, whereas in previous studies the analysis was typically performed at only one timepoint (see Supplementary Table S9). Additionally, the type of monitoring varied between studies, including visual observation, video monitoring, and EEG and vEEG monitoring. In addition, the latency from TBI, dose of PTZ, and the age and strain of the animals varied. Consequently, comparison of the outcome measures between the studies is challenging, as is the comparison of cross-sectional studies with the present follow-up study. However, consistent with previous cross-sectional studies, our vEEG-monitoring-based analysis revealed signs of increased seizure susceptibility within the TBI group at all monitoring timepoints.

Repeated administration of PTZ at a dose of 25 to 30 mg/kg can induce kindling in normal rats [64]. Hamm et al. [65] induced PTZ kindling in adult male Sprague-Dawley rats with a moderate central FPI via daily administration of PTZ at a dose similar to that used in the present study (25 mg/kg, intraperitoneally) for 24 d, starting 24 h after FPI. Interestingly, the TBI rats kindled at the same rate as the sham-operated controls. However, their Morris water-maze performance on D25–29 post-injury was better than that in unkindled TBI rats. We administered PTZ at a dose of 25 mg/kg four times over a period of 6 months, with the minimum interval being 30 d. To kindle a rat, the interval between PTZ administrations and the number of injections must be substantially higher than those in the present study. Moreover, 18% of the vEEG-monitored animals showed unprovoked seizures—which is less than the expected 25%—during the 6th post-TBI month [23,66]. Therefore, it is unlikely that the PTZ kindling effect contributed to increased seizure susceptibility in the animal cohort. Whether it affected epileptogenesis, resulting in lower prevalence of epilepsy than expected, remains to be further studied—particularly as some previous studies suggest that PTZ administration has a disease-modifying effect on epileptogenesis in a kainate-induced status epilepticus model [67]. Similarly, another proconvulsant—atipamezole—also had a favorable anti-epileptogenic effect in an electrical amygdala stimulation model of status epilepticus [68], as well as in the lateral FPI model [38].

The acute post-impact mortality in the present study was 15%, which was less than the anticipated 25% typical for severe TBI [23,69]. However, acute mortality can be reduced by careful post-impact care, which varies between laboratories [70], and therefore has a limited value as a justification criterion for “severe” TBI after FPI. Importantly, the average cortical lesion area in surviving rats at 6 months post-TBI was ~20 mm^2^, which is comparable to that in our studies with about 25% acute mortality [71], suggesting that the severity of brain injury corresponds to “severe TBI”. Previously, Bao et al. [41] reported that PTZ-induced seizures at 2 weeks post-injury exacerbated brain histopathology when assessed 3 days later. In the present study, the average area of the cortical lesion in injured rats with PTZ-induced seizures was smaller than that in rats without induced seizures, indicating no worsening of the cortical lesion due to repeated PTZ testing.

We previously demonstrated in a large EPITARGET animal cohort that the average weight loss during the first week after the median 3.3 atm impact was 12% [66]. In the same animal cohort, the average composite neuroscore on D2 was 8. In the present study cohort, the D2 weight loss was 13% and the average D2 composite neuroscore was 11, suggesting that the severity of TBI in the present study cohort with the aimed impact force of 3.0 atm was comparable to that in our previous cohorts.

## 5. Conclusions

In this study, we monitored the evolution of seizure susceptibility in a well-described rat model of PTE. Although rats with TBI showed indications and some evolution of reduced seizure susceptibility over the 6-month follow-up in the PTZ seizure-susceptibility test, the difference compared with the sham-operated experimental controls and progression over time were lower than anticipated. Nevertheless, some individual and combinatory PTZ test parameters showed promise as biomarkers of epileptogenesis, differentiating rats that developed epileptiform activity from those that did not. As we demonstrated previously, N3–REM transitions during sleep were associated with the occurrence of seizures and epileptiform activity. Consistent with this finding, sleep disturbance in rats with epileptiform activity or seizures after TBI manifested as a reduced duration of REM sleep periods and increased numbers of transitions, particularly from the N3 to awake stages, indicating sleep instability in this seizure-prone sleep period. Further analyses are warranted to assess the potential of sleep disturbance parameters as biomarkers of epileptogenesis in larger animal cohorts.

## Figures and Tables

**Table 1:** Seizure susceptibility in the PTZ test on day (D) 30, D60, D90, and D180 after lateral-fluid-percussion-induced traumatic brain injury (TBI) or sham operation.

Parameter *(General linear model)*	Group	Time of PTZ Test				Intragroup Statistics *(Friedman’s 2-way ANOVA)*
		D30	D60	D90	D180	
Latency to 1st spike	Sham (16)	220 ± 215 (16)	191 ± 340 (16)	278 ± 447 (16)	149 ± 141 (16)	*Friedman’s 2-way ANOVA p = 0.036* *Bonferroni corrected p > 0.05 (all)*
	TBI (28)	280 ± 432 (27)	204 ± 296 (28)	140 ± 85 (28) *(Cohen’s d 0.50)*	173 ± 179 (27)	*NS*
Latency to 1st ED	Sham (16)	316 ± 388 (16)	205 ± 341 (16)	359 ± 546 (16)	239 ± 230 (16)	*Friedman’s 2-way ANOVA p = 0.026* *D60–D90 Bonferroni-corrected p = 0.024*
	TBI (28)	480 ± 662 (27)	311 ± 422 (28)	176 ± 138 (28) *(Cohen’s d 0.53)*	227 ± 180 (27)	*Friedman’s 2-way ANOVA p = 0.015* *D30–D90 Bonferroni-corrected p = 0.011*
Latency to 1st seizure	Sham (16)	1192 ± 1374 (5)	197 ± 84 (5)	658 ± 1168 (7)	588 ± 853 (9)	*NS*
	TBI (28)	370 ± 209 (4) *(Cohen’s d 0.79)*	276 ± 148 (12)	177 ± 93 (15) *(Cohen’s d 0.75)*	326 ± 287 (16)	*Friedman’s 2-way ANOVA p = 0.044* *D30–D90 Bonferroni-corrected p = 0.037*
Number of spikes	Sham (16)	1342 ± 1127 (16)	1720 ± 1187 (16)	1260 ± 1743 (16)	1598 ± 1362 (16)	*NS*
	TBI (28)	1329 ± 812 (27)	1447 ± 1030 (28)	1600 ± 1404 (28)	1467 ± 1363 (27)	*NS*
Number of EDs	Sham (16)	157 ± 137 (16)	163 ±105 (16)	161 ± 112 (16)	139 ± 97 (16)	*NS*
	TBI (28)	176 ± 124 (27)	170 ± 132 (28)	175 ± 138 (28)	119 ± 98 (27)	*NS*
Number of seizures *(Time effect 0.000)*	Sham (16)	0.31 ± 0.48 (16)	0.38 ± 0.62 (16)	0.44 ± 0.51 (16)	1.06 ± 1.12 (16)	*Friedman’s 2-way ANOVA p = 0.041* *Bonferroni-corrected p > 0.05 (all)*
	TBI (28)	0.19 ± 0.48 (27)	0.82 ± 1.22 (28)	0.82 ± 0.91 (28)	1.15 ± 1.10 (27)	*Friedman’s 2-way ANOVA p = 0.000* *D30–D180 Bonferroni-corrected p = 0.006*
Duration of 1st seizure	Sham (16)	30 ± 7 (3)	58 ± 22 (5)	76 ± 43 (7)	53 ± 36 (9)	*NS*
	TBI (28)	64 ± 70 (4) *(Cohen’s d 0.62)*	64 ± 33 (12)	69 ± 32 (15)	76 ± 22 (16) *(Cohen’s d 0.82)*	*NS*

Data are shown as the mean ± standard deviation of the mean. Animal numbers are shown in parentheses. Abbreviations: D, day; ED, epileptiform discharge; NS, not significant; PTZ, pentylenetetrazol; TBI, traumatic brain injury. Statistical significance: time, group, and time x group effects were tested using a general linear model (left column in parentheses). Differences between timepoints within the sham or TBI groups were tested using related-samples Friedman’s 2-way ANOVA with Bonferroni correction for multiple testing (right column). No differences were detected between the sham and TBI groups at any testing point (Mann–Whitney U test). However, Cohen’s delta between the sham and TBI group, showed moderate (≥0.50) and large effect sizes (≥0.80) (in parentheses).

**Table 2:** Duration of waking and sleep periods during the lights-on and lights-off periods in different groups.

	Lights-Off (h)		Lights-On (h)		Lights-On and Lights-Off (h)	
Group	Sleep	Awake	Sleep	Awake	Sleep	Awake
Sham (11)	5.58 ± 0.89 ^##^	6.39 ± 0.92 **	9.04 ± 0.71	2.95 ± 0.71	14.62 ± 1.50 ^&&^	9.34 ± 1.53
TBI (14)	5.14 ± 0.57 ^##^	6.84 ± 0.57 **	9.00 ± 0.78	3.00 ± 0.78	14.14 ± 1.29 ^&&^	9.85 ± 1.11
TBIe− (7)	5.11 ± 0.49 ^#^	6.88 ± 0.49 *	9.11 ± 0.90	2.88 ± 0.90	14.22 ± 1.17 ^&^	9.76 ± 1.17
TBIe+ (7)	5.17 ± 0.68^#^	6.82 ± 0.67 *	8.88 ± 0.68	3.11 ± 0.69	14.05 ± 1.31 ^&^	9.93 ± 1.13
TBI− (11)	5.24 ± 0.56 ^##^	6.75 ± 0.56 **	9.10 ± 0.79	2.88 ± 0.79	14.34 ± 1.58 ^&&^	9.64 ± 1.16
TBI+ (3)	4.76 ± 0.52	7.21 ± 0.52	8.61 ± 0.75	3.39 ± 0.75	13.37 ± 0.42	10.61 ± 0.41

Data are shown as the mean ± standard deviation of the mean. Numbers of animals are in parentheses. Abbreviations: h, hour; TBI, traumatic brain injury; TBIe+, TBI rats with epileptiform activity; TBIe−, TBI rats without epileptiform activity; TBI+, TBI rats with epilepsy; TBI−, TBI rats without epilepsy. Statistical significance: ^#^, *p* < 0.05; ^##^, *p* < 0.01 compared with sleep duration during the lights-on period (Wilcoxon); *, *p* < 0.05; **, *p* < 0.01 compared with the waking period when the lights were on; ^&^, *p* < 0.05; ^&&^, *p* < 0.01 compared with the awake duration during the lights-on and lights-off periods (Wilcoxon). No differences were detected between the sham vs. TBI, TBIe+ vs. TBIe−, or TBI+ vs. TBI− groups (Mann–Whitney U test).

**Table 3:** Duration of different sleep–awake stages in sham-operated experimental controls and in rats with traumatic brain injury (TBI) during the lights-on and lights-off periods. A 24-h sleep EEG epoch was recorded in the 7th post-TBI month.

Parameter	Lights-On		Lights-Off		All	
	Sham	TBI	Sham	TBI	Sham	TBI
	(*n* = 11)	(*n* = 14)	(*n* = 11)	(*n* = 14)	(*n* = 11)	(*n* = 14)
	**Average duration of sleep periods (min)**				**Average lights-on and lights-off (min)**	
Wake	7.43 ± 4.07	6.53 ± 1.86	16.41 ± 5.03	15.32 ± 4.23	11.92 ± 3.46	10.93 ± 2.67
N2	1.53 ± 0.21	1.64 ± 0.32	1.16 ± 0.19	1.27 ± 0.28	1.34 ± 0.11	1.46 ± 0.27
N3	3.95 ± 0.91	3.53 ± 0.77	3.54 ± 0.64	3.07 ± 0.60	3.75 ± 0.73	3.31 ± 0.57
REM	2.17 ± 0.32	2.00 ± 0.27	1.91 ± 0.31	1.61 ± 0.21 *	2.04 ± 0.25	1.81 ± 0.15 *
	**Duration of sleep stages (h)**				**Total duration (h)**	
Wake	2.94 ± 0.71	2.99 ± 0.78	6.39 ± 0.92	6.85 ± 0.57	9.34 ± 0.53	9.84 ± 0.11
N2	1.02 ± 0.72	1.20 ± 0.75	0.48 ± 0.28	0.71 ± 0.45	1.49 ± 0.96	1.91 ± 1.12
N3	5.50 ± 0.82	5.30 ± 0.72	3.62 ± 0.67	3.19 ± 0.48	9.13 ± 1.39	8.49 ± 1.13
REM	2.53 ± 0.46	2.49 ± 0.73	1.47 ± 0.31	1.24 ± 0.38	3.99 ± 0.71	3.73 ± 1.05

Data are shown as the mean ± standard deviation of the mean. Statistical significance: * *p* < 0.05 compared with the sham group (Mann–Whitney U test). Abbreviations: N2, N2 sleep stage; N3, N3 sleep stage; REM, rapid eye movement sleep; TBI, traumatic brain injury; W, awake.

**Table 4:** Number of transitions and fragmentation index in sham-operated experimental controls and in rats with traumatic brain injury (TBI) during the lights-on and lights-off periods. A 24-h sleep EEG epoch was recorded on the 7th post-TBI month.

Parameter	Lights-On		Lights-Off		TOTAL	
	Sham	TBI	Sham	TBI	Sham	TBI
	(*n* = 11)	(*n* = 14)	(*n* = 11)	(*n* = 14)	(*n* = 11)	(*n* = 14)
**Number of transitions from a deeper to a lighter sleep stage**						
N2–Awake	4.09 ± 1.49	6.42 ± 1.82	5.54 ± 2.06	6.00 ± 1.20	9.63 ± 3.22	12.43 ± 2.76
N3–Awake	10.54 ± 1.60	8.07 ± 1.43	8.72 ± 1.09	10.50 ± 1.23	19.27 ± 2.38	18.57 ± 2.34
REM–Awake	11.91 ± 2.09	13.14 ± 1.33	10.09 ± 0.79	11.29 ± 0.74	22.00 ± 2.17	24.43 ± 1.36
N3–N2	9.27 ± 2.05	11.57 ± 2.85	5.81 ± 1.82	7.92 ± 1.64	15.09 ± 3.52	19.50 ± 4.17
REM–N2	13.18 ± 3.36	14.93 ± 3.33	4.91 ± 1.19	6.71 ± 1.54	18.09 ± 4.52	21.64 ± 4.71
REM–N3	47.64 ± 4.49	47.43 ± 6.51	32.55 ± 3.27	28.14 ± 4.21	80.18 ± 7.35	75.57 ± 10.22
Total	96.63 ± 4.35	101.57 ± 3.54	67.64 ± 4.36	70.57 ± 2.71	164.27 ± 7.94	172.14 ± 4.50
Deep-to-light sleep fragmentation index	8.01 ± 0.37	8.39 ± 0.35	5.73 ± 0.37	5.88 ± 0.22	6.83 ± 0.33	7.14 ± 0.21
**Number of transitions to**						
Awake	21.73 ± 3.07	26.50 ± 2.91	24.36 ± 1.98	28.36 ± 2.29	46.09 ± 4.30	54.86 ± 4.37
N2	29.82 ± 5.47	42.29 ± 6.73	26.18 ± 5.31	33.00 ± 5.09	56.00 ± 10.42	75.29 ± 10.91
N3	73.45 ± 2.66	86.50 ± 4.14 *	62.27 ± 3.65	63.29 ± 2.51	135.73 ± 5.53	149.79 ± 4.72
REM	59.73 ± 3.28	70.93 ± 4.72 *	48.55 ± 3.39	46.07 ± 3.56	108.27 ± 5.58	117.00 ± 7.19
Total	184.73 ± 9.88	226.21 ± 10.91 *	161.40 ± 10.72	170.71 ± 7.83	346.09 ± 18.39	396.93 ± 13.70 *
Fragmentation index	15.31 ± 0.81	18.67 ± 0.94 *	13.47 ± 0.89	14.23 ± 0.65	14.40 ± 0.94	16.46 ± 0.59 *

Data are shown as the mean ± standard deviation of the mean. Statistical significance: * *p* < 0.05 compared with the sham group (Mann–Whitney U test). Abbreviations: N2, N2 sleep stage; N3, N3 sleep stage; REM, rapid eye movement sleep; TBI, traumatic brain injury; W, awake.

**Table 5:** Pentylenetetrazol (PTZ) seizure susceptibility test and sleep parameters as combinatory biomarkers of prior traumatic brain injury (TBI) and post-traumatic epileptogenesis. Combination of PTZ test parameters performed moderately well in differentiating sham vs. TBI and TBIe+ vs. TBIe− animals, and survived the leave-one-out cross-validation. Parameters included in the combination biomarkers are specified in the footnote. Note that none of the individual PTZ test parameters differentiated the sham vs. TBI animals, but some of them showed sensitivity and specificity as biomarkers of epileptogenesis (Supplementary Table S7). Several individual sleep parameters showed sensitivity and specificity as biomarkers of prior TBI and epileptogenesis (Supplementary Table S8).

	ROC AUC (95% CI) without Cross-Validation	ROC AUC (95% CI) with Cross-Validation	Misclassification Rate	Sensitivity	Specificity	Precision
**PTZ seizure-susceptibility test parameters**						
Sham (16) vs. TBI (28)						
Combination of A–C D60 (16/28)	0.759 (0.578–0.887) **	0.679 (0.474–0.833)^0.05^	0.364	0.536	0.813	0.833
Combination of A–E D60 (5/12)	0.967 (0.750–1.000) ***	0.500 (0.050–0.869)	0.294	0.750	0.600	0.818
Combination of A–C D90 (16/28)	0.670 (0.492–0.816)^0.05^	0.526 (0.385–0.731)	0.546	0.464	0.438	0.591
Combination of A–E D90 (7/15)	0.914 (0.688–0.993) ***	0.733 (0.438–0.902) *	0.318	0.733	0.571	0.786
TBIe+ (7) vs. TBI− (15)						
Combination of A–C D90 (7/15)	0.895 (0.667–0.987) ***	0.743 (0.472–0.922)^0.05^	0.364	0.667	0.571	0.769
Combination of A–C D180 (7/15)	0.895 (0.653–0.992) ***	0.752 (0.483–0.929) *	0.318	0.733	0.571	0.786
Combination of A–E D180 (4/9)	0.889 (0.541–0.950) *	0.681 (0.350–1.000)	0.308	0.778	0.500	0.778
**TBI+ (4) vs. TBI− (18)**						
Combination of A–C D90 (4/18)	1.000 (0.990–1.000) **	0.653 (0.342–0.939)	0.227	0.500	0.833	0.400
Combination of A–C D180 (4/18)	0.861 (0.569–0.982) **	0.625 (0.000–0.950)	0.364	0.500	0.667	0.250
Combination of A–E D180 (4/9)	1.000 (0.983–1.000) ***	0.736 (0.429–1.000)	0.385	0.500	0.667	0.400
**Sleep disturbance parameters**						
Sham (16) vs. TBI (28)						
Combination of F–I (11/14)	0.896 (0.695–0.981) ***	0.792 (0.549–0.934) **	0.280	0.714	0.727	0.769
Combination of F–M (11/14)	0.948 (0.766–0.997) ***	0.591 (0.322–0.816)	0.360	0.636	0.643	0.583
TBIe+ (7) vs. TBIe− (15)						
Combination of F–I (7/7)	0.776 (0.411–0.979)^0.07^	0.367 (0.078–0.751)	0.500	0.571	0.429	0.500
Combination of F–M (7/7)	0.939 (0.849–0.970) ***	0.612 (0.325–0.844)	0.429	0.714	0.429	0.556
Combination of PTZ test and sleep: B, C, F-I D180 (7/7)	0.939 (0.849–0.970) ***	0.571 (0.269–0.847)	0.500	0.429	0.571	0.500
TBI+ (4) vs. TBI− (18)						
Combination of F–M (3/11)	0.970 (0.927–1.000) *	0.439 (0.182–0.885)	0.357	0.333	0.727	0.250

The number of animals in each analysis is in parentheses. The PTZ test parameters were obtained on D30, D60, D90, and D180 after TBI. Up to five PTZ test parameters were included in the combination biomarkers, and are referred as A–E: latency to the 1st ED (A), number of EDs (B), number of PTZ-induced seizures (C), latency to the 1st PTZ-induced seizure (D), and duration of the 1st PTZ-induced seizure €. Sleep disturbance was assessed on the 7th post-TBI month. Up to eight sleep disturbance parameters were included in the combination biomarkers, and are referred as F–M: average duration of REM episodes (lights-off) (F), number of transitions to N3 (lights-on) (G), total number of transitions (lights-on) (H), fragmentation index (lights-on) (I), average duration of REM periods (lights-on/off) (J), number of transitions to REM (lights-on) (K), total number of transitions (lights-on/off) (L), fragmentation index (lights-on/off) (M). Abbreviations: AUC, area under the curve; D, day; ROC, receiver operating characteristic analysis; CI, confidence interval; TBIe+, TBI rats with epileptiform activity; TBIe−, TBI rats without epileptiform activity; TBI+, TBI rats with epilepsy; TBIv, TBI rats without epilepsy. Statistical significance: ***, *p* < 0.001; **, *p* < 0.01; *, *p* < 0.05; *p* = 0.05 (with or without leave-one-out validation, MATLAB R2018b). The cross-validated misclassification rate, sensitivity, specificity, and precision were calculated for regression models that showed a cross-validated ROC AUC > 0.5 according to the statistical significance test.

## Data Availability

Not applicable.

## Supplementary Materials

The following are available online at https://www.mdpi.com/article/10.3390/biomedicines10051138/s1, Table S1: Seizure susceptibility in the PTZ test on day (D) 39, D60, D90, and D180 after lateral fluid-percussion-induced TBI in rats with (TBIe+) or without (TBIe− epileptiform activity. Table S2: Seizure susceptibility in the PTZ test on day (D) 30, D60, D90, and D180 after lateral fluid-percussion -induced TBI in rats with (TBI+) or without (TBI−) epilepsy. Table S3: Duration of different sleep–wake stages in rats with (TBIe+) or without any epileptiform activity (TBIe−) after traumatic brain injury (TBI) during the lights-on and lights-off periods. Table S4: Number of transitions and fragmentation index in rats with (TBIe+) or without any epileptiform activity (TBIe−) after traumatic brain injury (TBI) during the lights-on and lights-off periods. Table S5: Duration of different sleep–wake stages in rats with (TBI+) or without epilepsy (TBI−) after traumatic brain injury (TBI) during the lights-on and lights-off periods. Table S6: Number of transitions and fragmentation index in rats without (TBI−) or with epilepsy (TBI+) after traumatic brain injury (TBI) during the lights-on and lights-off periods. Table S7: Receiver operating characteristics (ROC) analysis of single pentylenetetrazol (PTZ)-test parameters as biomarkers for TBI and epileptogenesis. Table S8: Receiver operating characteristics (ROC) analysis of single sleep parameters as biomarkers for traumatic brain injury (TBI) and epileptogenesis. Table S9: Summary of studies assessing seizure susceptibility at different time points after lateral fluid-percussion -induced traumatic brain injury in rats.
