# Supplementary material for: Seizure Susceptibility and Sleep Disturbance as Biomarkers of Epileptogenesis after Experimental TBI
Source: Biomedicines. 2022 May 14;10(5):1138. doi: 10.3390/biomedicines10051138 (PMC9138230; doi:10.3390/biomedicines10051138)
Supplement: Supplementary file 1 [file biomedicines-10-01138-s001.zip › Supplementary Table S1.pdf]

Supplementary Table S1. Seizure susceptibility in the PTZ test on day (D) 39, D60, D90, and D180 after lateral fluid-percussion-induced TBI in rats with (TBIe+) or without (TBIe-) epileptiform activity.

**Parameter** *(General linear model)*	**Group**	**Time of PTZ test**				**Within Group Statistics** *(Friedman's 2-way ANOVA)*
		**D30**	**D60**	**D90**	**D180**	
Latency to 1st spike	TBIe- (7)	516 ± 700 (7)	126 ± 78 (7)	176 ± 98 (7	175 ± 126 (7)	*NS*
	TBIe+ (15)	221 ± 321 (14) *(Cohen's d 0.62)*	228 ± 383 (15)	130 ± 91 (15)	165 ± 222 (15)	*NS*
Latency to 1st ED *(Time effect p=0.007)*	TBIe- (7)	976 ± 833 (7)	277 ± 223 (7)	207 ± 71 (7)	316 ± 114 (7)	*Friedman's 2-way ANOVA p=0.027 D30-D90 Bonferroni corrected p=0.023*
	TBIe+ (15)	377 ± 599 (14) *(Cohen's d 0.88)*	240 ± 378 (15)	164 ± 180 (15)*	194 ± 215 (15)** *(Cohen's d 0.64)*	*NS*
Latency to 1st seizure *(Group effect p=0.002)*	TBIe- (7)	no seizures	341 ± 268 (2)	137 ± 60 (2)	486 ± 238 (4)	*NS*
	TBIe+ (15)	370 ± 209 (4)	233 ± 77 (8) *(Cohen's d 0.91)*	144 ± 87 (2)	287 ± 341 (9) *(Cohen's d 0.63)*	*Friedman's 2-way ANOVA p=0.044 D30-D90 Bonferroni corrected p=0.037*
Number of spikes	TBIe- (7)	925 ± 710 (7)	1409 ± 841 (7)	1821 ± 1036 (7)	971 ± 802 (7)	*NS*
	TBIe+ (15)	1439 ± 865 (14) *(Cohen's d 0.63)*	1514 ± 1174 (15)	1662 ± 1841 (15)	1697 ± 1316 (15) *(Cohen's d 0.61)*	*NS*
Number of EDs	TBIe- (7)	108 ± 92 (7)	167 ± 120 (7)	147 ± 125 (7)	104 ± 92 (7)	*NS*
	TBIe+ (15)	207 ± 137 (14) *(Cohen's d 0.80)*	182 ± 151 (15)	181 ± 140 (15)	137 ± 101 (15)	*NS*
Number of seizures *(Time effect p=0.003)*	TBIe- (7)	0 (7)	1.00 ± 1.92 (7)	0.29 ± 0.49 (7)	0.86 ± 0.90 (7)	*Friedman's 2-way ANOVA p=0.042 Bonferroni corrected p>0.05 (all)*
	TBIe+ (15)	0.36 ± 0.63 (14) *(Cohen's d 0.68)*	0.87 ± 0.99 (15)	0.93 ± 0.96 (15) *(Cohen's d 0.76)*	1.20 ± 1.15 (15)	*Friedman's 2-way ANOVA p=0.019 D30-D180 p=0.028 Bonferroni corrected p>0.05 (all)*
Duration of 1st seizure	TBIe- (7)	no seizures	51 ± 11 (2)	100 ± 34 (2)	86 ± 18 (4)	*NS*
	TBIe+ (15)	64 ± 70 (4)	60 ± 35 (8)	69 ± 35 (9) *(Cohen's d 0.90)*	73 ±24 (9) *(Cohen's d 0.56)*	*NS*

Data are shown as mean ± standard deviation of the mean. Animal numbers are shown in parentheses. ***Abbreviations***: D, day; ED, epileptiform discharge; PTZ, pentylenetetrazol; TBI, traumatic brain injury; TBIe+, TBI rats with epileptiform activity; TBIe-, TBI rats without epileptiform activity. ***Statistical significances***: Time, group and time * group effects were tested using general linear model (left column in parentheses). Differences between time-points within the TBIe+ and TBIe- groups were tested using related samples Friedman's two-way ANOVA with Bonferroni correction for multiple testing (right column). Differences between the TBIe+ and TBIe- groups at different time-points: ** p<0.01; * p < 0.05 (Mann-Whitney *U* test). Cohen's delta (d) between the TBIe+ and TBIe- groups is shown in parentheses for moderate (≥ 0.50) and large effect sizes (≥ 0.80).
