# Supplementary material for: Seizure Susceptibility and Sleep Disturbance as Biomarkers of Epileptogenesis after Experimental TBI
Source: Biomedicines. 2022 May 14;10(5):1138. doi: 10.3390/biomedicines10051138 (PMC9138230; doi:10.3390/biomedicines10051138)
Supplement: Supplementary file 1 [file biomedicines-10-01138-s001.zip › Supplementary Table S2 R1.pdf]

Supplementary Table S2. Seizure susceptibility in the PTZ test on day (D) 30, D60, D90, and D180 after lateral fluid-percussion -induced TBI in rats with (TBI+) or without (TBI-) epilepsy.

**Parameter** *(General linear model)*	**Group**	**Time of PTZ test**				**Within Group Statistics** *(Friedman's 2-way ANOVA)*
		**D30**	**D60**	**D90**	**D180**	
Latency to 1st spike	TBI- (18)	353 ± 517 (18)	190 ± 342 (18)	155 ± 101 (18)	176 ± 210 (18)	*NS*
	TBI+ (4)	118 ± 51 (3)	220 ± 219 (4)	99 ± 17 (4) *(Cohen's d 0.60)*	133 ± 95 (4)	*NS*
Latency to 1st ED	TBI- (18)	653 ± 758 (18)	258 ± 356 (18)	195 ± 169 (18)	253 ± 205 (18)	*Friedman's 2-way ANOVA p=0.001* *D30-D60 Bonferroni corrected p=0.040* *D30-D90 Bonferroni corrected p=0.000*
	TBI+ (4)	118 ± 51 (3) *(Cohen's d 0.75)*	226 ± 222 (4)	102 ± 21 (4) *(Cohen's d 0.60)*	143 ± 116 (4) *(Cohen's d 0.56)*	*NS*
Latency to 1st seizure	TBI- (18)	351 ± 115 (2)	273 ± 124 (8)	142 ± 89 (9)	325 ± 215 (9)	*NS*
	TBI+ (4)	389 ±341 (2)	182 ± 110 (2) *(Cohen's d 0.74)*	146 ± 23 (2)	400 ± 524 (4)	*NS*
Number of spikes *(Time * Group effect 0.035)*	TBI- (18)	1326 ± 873 (18)	1550 ± 1062 (18)	1213 ± 936 (18)	1376 ± 1022 (18)	*NS*
	TBI+ (4)	921 ±551 (3)	1168 ± 1143 (4)	3016 ± 2025 (4) *(Cohen's d 1.546)*	1872 ± 2005 (4)	*NS*
Number of EDs	TBI- (18)	186 ± 136 (18)	188 ± 141 (18)	147 ± 118 (18)	134 ± 103 (18)	*NS*
	TBI+ (4)	103 ± 55 (3) *(Cohen's d 0.64)*	127 ± 137 (4)	273 ± 168 (4) *(Cohen's d 0.99)*	90 ±57 (4)	*NS*
Number of seizures	TBI- (18)	0.17 ± 0.51 (18)	0.89 ± 1.37 (18)	0.78 ± 0.94 (18)	0.89 ± 1.02 (18)	*Friedman's 2-way ANOVA p=0.007* *D30-D180 p=0.024* *Bonferroni corrected p > 0.05 (all)*
	TBI+ (4)	0.67 ± 0.58 (3) *(Cohen's d 0.96)*	1.00 ± 1.16 (4)	0.50 ± 0.58 (4)	2.00 ± 0.82 (4)	*Friedman's 2-way ANOVA p=0.061*
Duration of 1st seizure	TBI- (18)	24 ± 14 (2)	53 ± 28 (8)	65 ± 32 (9)	84 ± 20 (9)	*NS*
	TBI+ (4)	103 ± 92 (2)	77 ± 48 (2)	115 ± 14 (2)	62 ±22 (4)	*NS*

Data are shown as mean ± standard deviation of the mean. Animal numbers are shown in parentheses. ***Abbreviations***: D, day; ED, epileptiform discharge; TBI, traumatic brain injury; TBI+, TBI rats with epilepsy; TBI-, TBI rats without epilepsy. ***Statistical significance***: Time, group, and time * group effects were tested using general linear model (left column in parentheses). Differences between time-points within the TBI+ and TBI- groups were tested using related samples Friedman's 2-way ANOVA with Bonferroni correction for multiple testing (right column). No differences were detected between the TBI+ and TBI- groups at any testing point (Mann-Whitney *U* test). Cohen's delta (d) between the TBI+ and TBI- groups is shown in parentheses for moderate (≥ 0.50) and large effect sizes (≥ 0.80).
