# Supplementary material for: Seizure Susceptibility and Sleep Disturbance as Biomarkers of Epileptogenesis after Experimental TBI
Source: Biomedicines. 2022 May 14;10(5):1138. doi: 10.3390/biomedicines10051138 (PMC9138230; doi:10.3390/biomedicines10051138)
Supplement: Supplementary file 1 [file biomedicines-10-01138-s001.zip › Supplementary Table S3.pdf]

Supplementary Table S3. Duration of different sleep–wake stages in rats with (TBIe+) or without any epileptiform activity (TBIe-) after traumatic brain injury (TBI) during the lights-on and lights-off periods. A 24-h sleep EEG epoch was recorded on the 7th post-TBI month.

Parameter	Lights-on		Lights-off		TOTAL	
	**TBIe- (*n* = 7)**	**TBIe+ (*n* = 7)**	**TBIe- (*n* = 7)**	**TBIe+ (*n* = 7)**	**TBIe- (*n* = 7)**	**TBIe+ (*n* = 7)**
	**Average duration of sleep periods (min)**				**Average Lights-on & Lights-off**	
**Wake**	6.99 ± 2.00	6.08 ± 1.73	16.37 ± 4.09	14.27 ± 4.42	11.68 ± 2.79	10.18 ± 2.54
**N2**	1.81 ± 0.29	1.48 ± 0.26	1.43 ± 0.30	1.12 ± 0.17*	1.63 ± 0.24	1.30 ± 0.19*
**N3**	3.24 ± 0.70	3.84 ± 0.76	2.75 ± 0.33	3.40 ± 0.66	2.99 ± 0.48	3.63 ± 0.51*
**REM**	2.07 ± 0.35	1.94 ± 0.17	1.66 ± 0.24	1.56 ± 0.18	1.86 ± 0.17	1.75 ± 0.10
	**Duration of sleep stages (h)**				**Total duration (h)**	
**Wake**	2.88 ± 0.90	3.11 ± 0.69	6.88 ± 0.49	6.82 ± 0.67	9.76 ± 0.17	9.93 ± 1.13
**N2**	1.19 ± 0.60	1.22 ± 0.92	0.81 ± 0.42	0.61 ± 0.49	2.00 ± 0.91	1.82 ± 1.37
**N3**	5.00 ± 0.57	5.60 ± 0.79	2.93 ± 0.17	3.45 ± 0.56	7.93 ± 0.68	9.05 ± 1.25
**REM**	2.92 ± 0.72	2.06 ± 0.46*	1.38 ± 0.35	1.11 ± 0.39	4.30 ± 1.01	3.17 ± 0.80

Data are shown as mean ± standard deviation of the mean. ***Statistical significance***: * $p < 0.05$ compared with the TBIe- group (Mann-Whitney *U* test).
Abbreviations: N2, N2 sleep stage; N3, N3 sleep stage; REM, rapid eye-movement sleep; TBI, traumatic brain injury; W, wake.
