# Supplementary material for: Seizure Susceptibility and Sleep Disturbance as Biomarkers of Epileptogenesis after Experimental TBI
Source: Biomedicines. 2022 May 14;10(5):1138. doi: 10.3390/biomedicines10051138 (PMC9138230; doi:10.3390/biomedicines10051138)
Supplement: Supplementary file 1 [file biomedicines-10-01138-s001.zip › Supplementary Table S4.pdf]

Supplementary Table S4. Number of transitions and fragmentation index in rats with (TBIe+) or without any epileptiform activity (TBIe-) after traumatic brain injury (TBI) during the lights-on and lights-off periods. A 24-h sleep EEG epoch was recorded on the 7th post-TBI month.

Parameter	Lights-on TBIe- (*n* = 7)	Lights-on TBIe+ (*n* = 7)	Lights-off TBIe- (*n* = 7)	Lights-off TBIe+ (*n* = 7)	TOTAL TBIe- (*n* = 7)	TOTAL TBIe+ (*n* = 7)
**Number of Transitions from a Deeper to a Lighter Sleep Stage**						
**N2-Wake**	5.57 ± 0.92	7.29 ± 3.64	6.71 ± 1.23	5.29 ± 2.16	12.29 ± 2.04	12.57 ± 5.38
**N3-Wake**	5.00 ± 1.13	11.14 ± 2.10*	8.14 ± 1.53	12.86 ± 1.56*	13.14 ± 2.41	24.00 ± 2.86*
**REM-Wake**	12.14 ± 1.24	14.14 ± 2.42	11.14 ± 0.94	11.43 ± 1.23	23.29 ± 1.04	25.57 ± 2.55
**N3-N2**	8.29 ± 2.64	14.86 ± 4.98	7.86 ± 2.51	8.00 ± 2.34	16.14 ± 4.75	22.86 ± 7.01
**REM-N2**	13.14 ± 4.74	16.71 ± 4.96	6.43 ± 1.89	7.00 ± 2.60	19.57 ± 6.38	23.71 ± 7.37
**REM-N3**	58.43 ± 10.22	36.43 ± 6.24	32.14 ± 5.14	24.14 ± 6.71	90.57 ± 14.55	60.57 ± 12.90
**Total**	102.57 ± 5.04	100.57 ± 5.36	72.43 ± 3.09	68.71 ± 4.61	175.00 ± 7.30	169.29 ± 5.69
**Deep to Light Sleep Fragmentation Index**	8.65 ± 0.56	8.15 ± 0.45	6.04 ± 0.26	5.73 ± 0.38	7.33 ± 0.36	6.96 ± 0.25
**Number of Transitions to**						
**Wake**	22.00 ± 3.09	31.00 ± 4.53	26.57 ± 2.67	30.14 ± 3.83	48.57 ± 4.74	61.14 ± 6.89
**N2**	37.29 ± 6.83	47.29 ± 11.90	34.29 ± 5.99	31.71 ± 8.73	71.57 ± 11.94	79.00 ± 19.20
**N3**	88.71 ± 6.83	84.29 ± 5.10	64.57 ± 2.88	62.00 ± 4.31	153.29 ± 9.11	146.29 ± 3.13
**REM**	79.29 ± 6.81	62.57 ± 5.20	49.57 ± 4.79	42.57 ± 5.30	128.86 ± 10.47	105.14 ± 8.24
**Total**	227.29 ± 14.42	225.14 ± 17.55	175.00 ± 7.89	166.43 ± 14.06	402.29 ± 20.26	371.57 ± 19.84
**Fragmentation Index**	19.14 ± 1.42	18.19 ± 1.33	14.59 ± 0.66	13.88 ± 1.17	16.84 ± 0.93	16.08 ± 0.80

Data are shown as mean ± standard error of the mean. ***Statistical significance:*** * $p < 0.05$ compared with the TBIe- group (Mann-Whitney *U* test). ***Abbreviations:*** N2, N2 sleep stage; N3, N3 sleep stage; REM, rapid eye-movement sleep; TBI, traumatic brain injury; W, wake.
