# Supplementary material for: Seizure Susceptibility and Sleep Disturbance as Biomarkers of Epileptogenesis after Experimental TBI
Source: Biomedicines. 2022 May 14;10(5):1138. doi: 10.3390/biomedicines10051138 (PMC9138230; doi:10.3390/biomedicines10051138)
Supplement: Supplementary file 1 [file biomedicines-10-01138-s001.zip › Supplementary Table S5.pdf]

Supplementary Table S5. Duration of different sleep–wake stages in rats with (TBI+) or without epilepsy (TBI-) after traumatic brain injury (TBI) during the lights-on and lights-off periods. A 24-h sleep EEG epoch was recorded on the 7th post-TBI month.

**Parameter**	**Lights-on**		**Lights-on**		**TOTAL**	
	**TBI- (*n* = 11)**	**TBI+ (*n* = 3)**	**TBI- (*n* = 3)**	**TBI+ (*n* = 3)**	**TBI- (*n* = 11)**	**TBI+ (*n* = 3)**
	**Average duration of sleep periods (min)**				**Average Lights-on & Lights-off**	
**Wake**	6.74 ± 2.05	5.78 ± 0.65	15.00 ± 4.49	16.52 ± 3.55	10.86 ± 2.94	11.15 ± 1.80
**N2**	1.71 ± 0.29	1.39 ± 0.33	1.34 ± 0.27	1.05 ± 0.23	1.53 ± 0.24	1.22 ± 0.27
**N3**	3.47 ± 0.77	3.78 ± 0.84	2.86 ± 0.36	3.86 ± 0.73*	3.16 ± 0.50	3.82 ± 0.60
**REM**	2.06 ± 0.27	1.78 ± 0.09	1.61 ± 0.20	1.62 ± 0.27	1.84 ± 0.14	1.70 ± 0.13
	**Duration of sleep stages (h)**				**Total duration (h)**	
**Wake**	2.89 ± 0.79	3.39 ± 0.75	6.75 ± 0.56	7.21 ± 0.52	9.64 ± 1.16	10.61 ± 0.41
**N2**	1.26 ± 0.76	0.99 ± 0.83	0.81 ± 0.44	0.31 ± 0.25	2.08 ± 1.12	1.30 ± 1.08
**N3**	5.18 ± 0.76	5.77 ± 0.44	3.10 ± 0.43	3.51 ± 0.61	8.28 ± 1.13	9.27 ± 0.88
**REM**	2.66 ± 0.73	1.85 ± 0.13	1.33 ± 0.39	0.95 ± 0.20	3.99 ± 1.04	2.80 ± 0.28

Data are shown as mean ± standard deviation of the mean. ***Statistical significance***: * $p < 0.05$ compared with the TBI- group (Mann-Whitney *U* test).
Abbreviations: N2, N2 sleep stage; N3, N3 sleep stage; REM, rapid eye-movement sleep; TBI, traumatic brain injury; W, wake.
