# Supplementary material for: Seizure Susceptibility and Sleep Disturbance as Biomarkers of Epileptogenesis after Experimental TBI
Source: Biomedicines. 2022 May 14;10(5):1138. doi: 10.3390/biomedicines10051138 (PMC9138230; doi:10.3390/biomedicines10051138)
Supplement: Supplementary file 1 [file biomedicines-10-01138-s001.zip › Supplementary Table S6.pdf]

Supplementary Table S6. Number of transitions and fragmentation index in rats without (TBI-) or with epilepsy (TBI+) after traumatic brain injury (TBI) during the lights-on and lights-off periods. A 24-h sleep EEG epoch was recorded on the 7th post-TBI month.

Parameter	Lights-on		Lights-off		TOTAL	
	TBI- (*n* = 11)	TBI+ (*n* = 3)	TBI- (*n* = 11)	TBI+ (*n* = 3)	TBI- (*n* = 11)	TBI+ (*n* = 3)
**Number of Transitions from a Deeper to a Lighter Sleep Stage**						
**N2-Wake**	7.18 ± 2.24	3.67 ± 2.03	7.18 ± 1.30	1.67 ± 1.20	14.36 ± 3.21	5.33 ± 3.18
**N3-Wake**	6.18 ± 1.23	15.00 ± 2.00*	9.64 ± 1.34	13.67 ± 2.67	15.82 ± 2.16	28.67 ± 4.06*
**REM-Wake**	12.45 ± 1.18	15.67 ± 4.98	11.82 ± 0.87	9.33 ± 0.67	24.27 ± 1.18	25.00 ± 5.51
**N3-N2**	9.45 ± 2.55	19.33 ± 9.53	7.73 ± 1.74	8.67 ± 5.17	17.18 ± 3.98	28.00 ± 13.86
**REM-N2**	14.00 ± 3.70	18.33 ± 8.84	6.82 ± 1.74	6.33 ± 4.10	20.82 ± 5.28	24.67 ± 12.60
**REM-N3**	51.82 ± 7.80	47.43 ± 6.51	30.55 ± 5.06	19.33 ± 4.33	82.36 ± 12.17	50.67 ± 7.84
**Total**	101.09 ± 3.77	103.33 ± 10.91	73.73 ± 2.70	59.00 ± 2.31*	174.82 ± 4.92	162.33 ± 10.67
**Deep to Light Sleep Fragmentation Index**	8.41 ± 0.40	8.34 ± 0.92	6.15 ± 0.22	4.92 ± 0.19**	7.28 ± 0.24	6.65 ± 0.46
**Number of Transitions to**						
**Wake**	24.36 ± 3.07	34.33 ± 6.77	29.09 ± 2.82	25.67 ± 3.18	53.45 ± 5.05	60.00 ± 9.85
**N2**	41.45 ± 6.85	45.33 ± 22.58	36.91 ± 5.79	18.67± 7.88	78.36 ± 11.93	64.00 ± 30.37
**N3**	85.36 ± 4.90	90.67 ± 8.29	65.45 ± 2.55	55.33 ± 5.70	150.82 ± 5.99	146.00 ± 3.21
**REM**	73.27 ± 5.74	62.33 ± 4.98	49.10 ± 4.08	35.00 ± 1.53	122.36 ± 8.37	97.00 ± 6.33
**Total**	224.45 ± 11.89	232.67 ± 31.57	180.55 ± 7.49	134.67 ± 2.33**	405.00 ± 14.99	367.33 ± 31.88
**Fragmentation Index**	18.65 ± 1.07	18.74 ± 2.44	15.05 ± 0.62	11.23 ± 0.19	16.85 ± 0.66	15.05 ± 1.28

Data are shown as mean ± standard error of the mean. ***Statistical significance:*** * p < 0.05, ** p<0.01 compared with the TBI- group (Mann-Whitney *U* test). ***Abbreviations:*** N2, N2 sleep stage; N3, N3 sleep stage; REM, rapid eye-movement sleep; TBI, traumatic brain injury; W, wake.
