# Supplementary material for: Seizure Susceptibility and Sleep Disturbance as Biomarkers of Epileptogenesis after Experimental TBI
Source: Biomedicines. 2022 May 14;10(5):1138. doi: 10.3390/biomedicines10051138 (PMC9138230; doi:10.3390/biomedicines10051138)
Supplement: Supplementary file 1 [file biomedicines-10-01138-s001.zip › Supplementary Table S7.pdf]

Supplementary Table S7. Receiver operating characteristics (ROC) analysis of single pentylenetetrazol (PTZ)-test parameters as biomarkers for TBI and epileptogenesis. The PTZ test was performed on day (D) 30, D60, D90, and D180. The TBI, TBIe+, and TBI+ groups were labeled as "state variables" in the corresponding ROC analysis in SPSS.

Parameter	Sham *vs.* TBI		TBIe- *vs.* TBIe+		TBI+ *vs.* TBI-	
	Nr Sham/TBI	AUC (95% CI)	Nr TBIe-/TBIe+	AUC (95% CI)	Nr TBI+/TBI-	AUC (95% CI)
**Latency to the 1st spike**						
D30	16/27	0.472	7/14	0.561	3/18	0.630
D60	16/28	0.546	7/15	0.519	4/18	0.681
D90	16/28	0.415	7/15	0.686	4/18	0.639
D180	16/27	0.530	7/15	0.562	4/18	0.542
**Latency to the 1st ED**						
D30	16/27	0.557	7/14	0.760* (0.516-1.004)	3/18	0.815* (0.567-1.062)
D60	16/28	0.614	7/15	0.757	4/18	0.514
D90	16/28	0.374	7/15	0.771* (0.547-0.969)	4/18	0.722
D180	16/27	0.517	7/15	0.857* (0.694-1.021)	4/18	0.764
**Latency to the 1st seizure**						
D30	5/4	0.400	0/4	na	2/2	0.500
D60	5/12	0.700	2/8	0.562	2/8	0.688
D90	7/15	0.324	2/9	0.500	2/9	0.788* (0.506-1.049)
D180	9/16	0.413	4/9	0.750	4/9	0.639
**Number of spikes**						
D30	16/27	0.520	7/14	0.724	3/18	0.593
D60	16/28	0.446	7/15	0.524	4/18	0.639
D90	16/28	0.446	7/15	0.590	4/18	0.792
D180	16/27	0.417	7/15	0.676	4/18	0.542
**Number of EDs**						
D30	16/27	0.550	7/14	0.724	3/18	0.704
D60	16/28	0.519	7/15	0.524	4/18	0.674
D90	16/28	0.553	7/15	0.600	4/18	0.806
D180	16/27	0.455	7/15	0.581	4/18	0.625

**Number of seizures**						
D30	16/27	0.424	7/14	0.643	3/18	0.759
D60	16/28	0.586	7/15	0.571	4/18	0.556
D90	16/28	0.604	7/15	0.695	4/18	0.556
D180	16/27	0.522	7/15	0.581	4/18	0.792** (0.586-0.997)
**Duration of the 1st seizure**						
D30	3/4	0.667	0/4	na	3/4	0.667
D60	5/12	0.575	2/8	0.531	5/12	0.575
D90	7/15	0.457	2/9	0.667	7/15	0.457
D180	9/16	0.719	4/9	0.569	9/16	0.719
**Cumulative seizure duration**						
D30	16/27	0.487	7/14	0.643	3/18	0.796
D60	16/28	0.581	7/15	0.567	4/18	0.562
D90	16/28	0.576	7/15	0.638	4/18	0.569
D180	16/27	0.531	7/15	0.505	4/18	0.639
**Racine score of the 1st seizure**						
D30	5/4	0.425	0/4	na	2/2	0.750
D60	5/12	0.333	2/8	0.719	2/8	0.719
D90	7/15	0.410	2/9	0.694	2/9	0.694
D180	9/16	0.486	4/9	0.611	4/9	0.792

Abbreviations: AUC, area under the curve; CI, confidence interval; D, day; ED, epileptiform discharge; na, not analyzed; Nr, number of rats in each analysis; ROC, receiver operating characteristic analysis; TBI, traumatic brain injury; TBIe+, rats with epileptiform activity; TBIe-, rats without any epileptiform activity; TBI+, rats with epilepsy, TBI-, rats without epilepsy. ***Statistical significance***: *, p<0.05; **, p<0.01 (ROC analysis in SPSS).
