# Supplementary material for: Seizure Susceptibility and Sleep Disturbance as Biomarkers of Epileptogenesis after Experimental TBI
Source: Biomedicines. 2022 May 14;10(5):1138. doi: 10.3390/biomedicines10051138 (PMC9138230; doi:10.3390/biomedicines10051138)
Supplement: Supplementary file 1 [file biomedicines-10-01138-s001.zip › Supplementary Table S8.pdf]

Supplementary Table S8. Receiver operating characteristics (ROC) analysis of single sleep parameters as biomarkers for traumatic brain injury (TBI) and epileptogenesis. Sleep was analyzed in a 24-h epoch sampled on the 7th post-TBI month. The TBI, TBIe+, and TBI+ groups were labeled as "state variables" in the corresponding ROC analysis.

Parameter	Sham *vs.* TBI		TBIe- *vs.* TBIe+		TBI+ *vs.* TBI-	
	Nr Sham/TBI	AUC (95% CI)	Nr TBIe-/TBIe+	AUC (95% CI)	Nr TBI+/TBI-	AUC (95% CI)
**Average duration of sleep periods (min)**						
**Lights-on**						
Wake	11/14	0.503	7/7	0.367	3/11	0.318
N2	11/14	0.640	7/7	0.245	3/11	0.258
N3	11/14	0.373	7/7	0.694	3/11	0.576
REM	11/14	0.347	7/7	0.449	3/11	0.182* (-0.061-0.424)
**Lights-off**						
Wake	11/14	0.468	7/7	0.367	3/11	0.606
N2	11/14	0.620	7/7	0.143** (-0.088-0.374)	3/11	0.167** -0.061-0.424)
N3	11/14	0.308	7/7	0.796* (0.554-1.038)	3/11	0.939*** (0.801-1.077)
REM	11/14	0.250* (0.047-0.453)	7/7	0.398	3/11	0.470
**Lights-on & Lights-off**						
Wake	11/14	0.425	7/7	0.388	3/11	0.576
N2	11/14	0.643	7/7	0.143** (-0.088-0.374)	3/11	0.182* (-0.061-0.424)
N3	11/14	0.344	7/7	0.837** (0.622-1.051)	3/11	0.879*** (0.684-1.074)
REM	11/14	0.227* (0.021-0.434)	7/7	0.286	3/11	0.212
**Average duration of sleep stages (h)**						
**Lights-on**						
Wake	11/14	0.513	7/7	0.551	3/11	0.636
N2	11/14	0.558	7/7	0.510	3/11	0.424
N3	11/14	0.416	7/7	0.714	3/11	0.818** (0.579-1,057)
REM	11/14	0.464	7/7	0.163** (-0.064-0.390)	3/11	0.152** (-0.054-0.357)

**Lights-off**						
Wake	11/14	0.669	7/7	0.490	3/11	0.727
N2	11/14	0.649	7/7	0.327	3/11	0.121***<br>(-0.074-0.316)
N3	11/14	0.305	7/7	0.806*<br>(0.570-1.043)	3/11	0.833**<br>(0.608-1.059)
REM	11/14	0.354	7/7	0.286	3/11	0.182**<br>(-0.039-0.403)
**Lights-on & Lights-off**						
Wake	11/14	0.623	7/7	0.571	3/11	0.788*<br>(0.545-1.031)
N2	11/14	0.591	7/7	0.449	3/11	0.333
N3	11/14	0.370	7/7	0.776*<br>(0.521-1.030)	3/11	0.788*<br>(0.545-1.031)
REM	11/14	0.432	7/7	0.204*<br>(-0.049-0.457)	3/11	0.182**<br>(-0.046-0.410)
**Number of Transitions from a Deeper to a Lighter Sleep Stage**						
**Lights-on**						
N2-Wake	11/14	0.630	7/7	0.418	3/11	0.318
N3-Wake	11/14	0.357	7/7	0.827**<br>(0.598-1.055)	3/11	0.970***<br>(0.882-1.057)
REM-Wake	11/14	0.584	7/7	0.592	3/11	0.621
N3-N2	11/14	0.529	7/7	0.622	3/11	0.682
REM-N2	11/14	0.513	7/7	0.592	3/11	0.545
REM-N3	11/14	0.461	7/7	0.224*<br>(-0.026-0.475)	3/11	0.318
Total	11/14	0.568	7/7	0.531	3/11	0.576
**Lighs-off**						
N2-Wake	11/14	0.584	7/7	0.378	3/11	0.136***<br>(-0.065-0.338)
N3-Wake	11/14	0.591	7/7	0.847**<br>(0.631-1.063)	3/11	0.818**<br>(0.579-1.057)
REM-Wake	11/14	0.597	7/7	0.510	3/11	0.227*<br>(-0.030-0.458)
N3-N2	11/14	0.649	7/7	0.561	3/11	0.515
REM-N2	11/14	0.607	7/7	0.480	3/11	0.470
REM-N3	11/14	0.406	7/7	0.347	3/11	0.303

Total	11/14	0.568	7/7	0.367	3/11	0.030*** (-0.059-0.120)
**Lights-on & Lights-off**						
N2-Wake	11/14	0.633	7/7	0.388	3/11	0.182* (-0.061-0.424)
N3-Wake	11/14	0.481	7/7	0.857** (0.651-1.063)	3/11	0.909*** (0.742-1.077)
REM-Wake	11/14	0.620	7/7	0.643	3/11	0.485
N3-N2	11/14	0.539	7/7	0.592	3/11	0.621
REM-N2	11/14	0.536	7/7	0.541	3/11	0.545
REM-N3	11/14	0.422	7/7	0.245	3/11	0.303
Total	11/14	0.597	7/7	0.510	3/11	0.333
**Fragmentation**						
Deep to Light Sleep Fragmentation Lights-on	11/14	0.571	7/7	0.429	3/11	0.545
Deep to Light Sleep Fragmentation Lights-off	11/14	0.532	7/7	0.357	3/11	0.000*** (0.000-0.000)
Deep to Light Sleep Fragmentation Lights-on & Lights-off	11/14	0.591	7/7	0.80	3/11	0.348
**Number of Transitions to**						
**Lights-on**						
Wake	11/14	0.610	7/7	0.745	3/11	0.742
N2	11/14	0.649	7/7	0.622	3/11	0.576
N3	11/14	0.766** (0.573-0.960)	7/7	0.459	3/11	0.591
REM	11/14	0.740* (0.543-0.937)	7/7	0.204* (-0.049-0.457)	3/11	0.242* (-0.006-0.491)
Total	11/14	0.786** (0.605-0.967)	7/7	0.490	3/11	0.500
**Lights-off**						
Wake	11/14	0.643	7/7	0.622	3/11	0.455
N2	11/14	0.604	7/7	0.469	3/11	0.242
N3	11/14	0.513	7/7	0.439	3/11	0.258
REM	11/14	0.419	7/7	0.306	3/11	0.167** (-0.046-0.491)
Total	11/14	0.591	7/7	0.388	3/11	0.000*** (0.000-0.000)
**Lights-on & Lights-off**						
Wake	11/14	0.659	7/7	0.714	3/11	0.652
N2	11/14	0.643	7/7	0.571	3/11	0.424

N3	11/14	0.692	7/7	0.439	3/11	0.424
REM	11/14	0.617	7/7	0.204* (-0.049-0.457)	3/11	0.182* (-0.046-0.410)
Total	11/14	0.747* (0.542-0.953)	7/7	0.449	3/11	0.,273
**Fragmentation**						
Fragmentation index Lights-on	11/14	0.769** (0.582-0.957)	7/7	0.449	3/11	0.485
Fragmentation index Lights-off	11/14	0.591	7/7	0.388	3/11	0.000*** (0.000-0.000)
Fragmentation index Lights-on & Lights-off	11/14	0.740* (0.531-0.949)	7/7	0.408	3/11	0.212

Abbreviations: AUC, area under the curve; CI, confidence interval; D, day; ED, epileptiform discharge; Nr, number of rats in each analysis; na, not analyzed; ROC, receiver operating characteristic analysis; TBI, traumatic brain injury; TBIe+, rats with epileptiform activity; TBIe-, rats without any epileptiform activity; TBI+, rats with epilepsy, TBI-, rats without epilepsy. ***Statistical significance***: *, p<0.05; **, p<0.01, ***p<0.001 (ROC analysis in SPSS).
