# Supplementary material for: Seizure Susceptibility and Sleep Disturbance as Biomarkers of Epileptogenesis after Experimental TBI
Source: Biomedicines. 2022 May 14;10(5):1138. doi: 10.3390/biomedicines10051138 (PMC9138230; doi:10.3390/biomedicines10051138)
Supplement: Supplementary file 1 [file biomedicines-10-01138-s001.zip › Supplementary Table S9.pdf]

Supplementary Table S9. Summary of studies assessing seizure susceptibility at different time points after lateral fluid-percussion -induced traumatic brain injury in rats. Studies are organized from the shortest to the longest post-injury assessment time point. ***Abbreviations:*** atm, atmospheric pressure; C, control; ED, epileptiform discharge; EEG, electroencephalogram; FPI, fluid-percussion injury; i.p., intraperitoneal; Nr, number; NS, not significant; PTZ, pentylenetetrazol; Sz, seizure; TBI, traumatic brain injury; wk, week; ⇧, increase; ⇩, decrease.

**Reference**	**Strain**	**Sex (C/TBI)**	**Age at TBI**	**FPI (atm)**	**PTZ test**			**Outcome measures**											
					Time after TBI	Dose (route)	Monitoring	Latency to 1st spike	Latency to 1st ED	Latency to 1st Sz	Nr of spikes	Nr of EDs	Nr of Sz	ED duration	% of rats with Sz	Sz duration	Racine score	PTZ dose needed to induce sz	Mortality
(Saraiva et al., 2012)[21]	Wistar	Male (7-9 per group)	Adult	Lateral (3.5)	4 d	35 mg/kg (i.p.)	Visual observation, EEG for 20 min			⇩						⇧	⇧		
(Bao et al., 2012)[54]	Sprague-Dawley	Male (10/10)	Adult	Lateral (1.9-2.1)	2 wk	30 mg/kg (i.p.)	Visual observation for 1 h						⇧			⇧	⇧		
(Silva et al., 2011)[43]	Wistar	Male (8-12 per group)	Adult	Lateral (4.1)	5 wk	30 mg/kg (i.p.)	Visual observation, EEG for 20 min			⇩						⇧	⇧		
(Silva et al., 2013)[44]	Wistar	Male (8-11 per group)	Adult	Lateral (4.1)	5 wk	35 mg/kg (i.p.)	Visual observation, EEG for 20 min			⇩						⇧	⇧		
(Gerbatin et al., 2019)[42]	Wistar	Male (8-9 per group)	Adult	Lateral (3.8)	5 wk	35 mg/kg (i.p.)	Visual observation, EEG for 20 min			⇩						⇧	⇧		
(Wang et al., 2016)[55]	Sprague-Dawley	Male (10/10)	Adult	Lateral (2.8-3.3)	6 wk	65 mg/kg (i.p.)	Video-EEG (60 min)			⇩									90% in the TBI group
(Smith et al., 2018)[45]	Wistar	Male (8/10)	Adult	Lateral (2.8-3.2)	6 wk	65mg/kg (i.p.)	Observation, video-monitoring (30 min)											⇩	
(Atkins et al., 2010)[47]	Sprague-Dawley	Male (17/16)	Adult	Lateral (1.8-2.2)	12 wk	30 mg/kg (i.p.)	Visual observation for 1 h			NS			⇧				NS		
(Huusko et al., 2015)[36]	Sprague-Dawley	Male (6/9)	Adult	Lateral (3.3)	5 months	25 mg/kg (i.p.)	Video-EEG (60 min)	⇩	NS		NS	⇧		NS	NS				
(Nissinen et al., 2017)[38]	Sprague-Dawley	Male (16/35)	Adult	Lateral (3.3-3.4)	5 months	25 mg/kg (i.p.)	Video-EEG (60 min)	⇩	NS	NS	NS	NS			⇧				
(Wang et al., 2021)[11]	Sprague-Dawley	Male (5/11)	Adult	Lateral (3.2)	23 wk	25 mg/kg (i.p.)	Video-EEG (120 min)	NS	NS	NS					NS	NS	NS		
(Yasmin et al., 2019)[56]	Sprague-Dawley	Male (716)	Adult	Lateral (3.3)	7 months	25 mg/kg (i.p.)	Video-EEG (120 min)	NS		NS						NS			
(Hayward et al., 2010)[34]	Sprague-Dawley	Male (4/10)	Adult	Lateral (3.2-3.4)	9 months	25 mg/kg (i.p)	Visual observation, video-EEG (60 min)	⇩				⇧							
(van Vliet et al., 2020)[57]	Sprague-Dawley	Male (6/12)	Adult	Lateral (3.0)	11 months	25 mg/kg (i.p.)	Observation, video-EEG (60 min)	NS			⇧				NS				
(Kharatishvili et al., 2007)[58]	Sprague-Dawley	Male (6/9)	Adult	Lateral (2.3-3.2)	12 months	30 mg/kg (i.p.)	Visual observation, video-EEG (60 min)	⇩		NS	⇧	⇧				NS	NS		
(Kharatishvili et al., 2007)[58]	Sprague-Dawley	Male (10/14)	Adult	Lateral (2.3-3.2)	12 months	25 mg/kg (i.p.)	Visual observation, video-EEG (60 min)	⇩		NS	⇧	⇧				NS	NS		
